# Thalamic remodeling underlying below-level neuropathic pain following spinal cord injury: neuroimmune, neurochemical, and circuit-level mechanisms

**DOI:** 10.3389/fncel.2026.1969437

**Published:** 2026-10-29

**Authors:** Wenguang Chen, Yurou Ai, Baoxiu Yi, Cong Yu, Ensi Hong

**Affiliations:** 1 College of Clinical Medicine, Jiangxi University of Traditional Chinese Medicine, Nanchang, China; 2 The Affiliated Hospital of Jiangxi University of Traditional Chinese Medicine, Nanchang, China; 3 East China University of Technology, Nanchang, China

**Keywords:** below-level neuropathic pain, neural circuits, neurochemistry, neuroimmune signaling, spinal cord injury, thalamus

## Abstract

Below-level neuropathic pain (BLNP) is a common and refractory complication of spinal cord injury (SCI). Its persistence and significant clinical heterogeneity are difficult to explain solely through mechanisms within the injured spinal cord. The thalamus receives ascending sensory input from the spinal cord and has extensive connections with the somatosensory cortex, insula, and cingulate cortex; sustained thalamic remodeling may serve as a critical mechanism by which abnormal spinal activity drives central pain processing. Existing evidence indicates that partial sensory deafferentation and abnormal neural activity transmitted via spared pathways can alter thalamic afferent input, while signaling involving C-C motif chemokine ligand 21 (CCL21) may influence the local neuroimmune environment of the thalamus. Microglial responses, altered excitatory-inhibitory synaptic regulation, changes in ion-channel expression, and synaptic structural modifications are all associated with hyperexcitability and aberrant firing patterns in thalamic neurons. These local changes may also be accompanied by the reorganization of corticothalamic feedback and alterations in thalamocortical functional connectivity. Human imaging studies suggest that pain phenotypes are associated with thalamic metabolism, related white-matter microstructure, and functional connectivity. This review examines thalamic afferent status, neuroimmune signaling, neurochemical and electrophysiological changes, and the reorganization of related circuits to enhance understanding of supraspinal mechanisms, systematically consolidate existing evidence, and provide a basis for investigating thalamus-related candidate clinical measures and mechanism-based interventions.

## 1 Introduction

Neuropathic pain is a common and disabling complication of SCI, persistently affecting daily activities, sleep, mood, social participation, and rehabilitation. A systematic review and meta-analysis estimated the pooled prevalence of chronic pain and neuropathic pain post-SCI at approximately 68% and 58%, respectively, despite considerable heterogeneity among studies (Hunt et al., 2021). Another updated meta-analysis applying established pain-classification criteria included 24 studies and 6,318 participants. Results showed a pooled prevalence of 57% for neuropathic pain post-SCI, with BLNP at approximately 30%; subgroup analysis found no clear association between its prevalence and injury severity (Salehian et al., 2025). The International Spinal Cord Injury Pain Classification categorizes SCI-related pain into nociceptive and neuropathic pain, with the latter further subdivided into at-level, below-level, or other neuropathic pain subtypes. BLNP refers to neuropathic pain attributed to damage within the spinal somatosensory system and perceived across three or more dermatomes below the neurological level of injury. It may occur with or without corresponding sensory loss and cannot be diagnosed based solely on the location of pain (Bryce et al., 2012). BLNP may manifest as spontaneous or stimulus-evoked burning, electric shock-like, pricking, or cold pain, often accompanied by mechanical allodynia, hyperalgesia, numbness, and reduced thermal or pinprick sensation. Longitudinal studies indicate that pain may emerge within months after injury, and that its type, intensity, and impact on daily life vary over time rather than directly corresponding to the neurological level of injury or severity (Siddall et al., 1999, 2003; Bryce et al., 2026). Overall efficacy provided by current pharmacological treatments, rehabilitation, and neuromodulation remains limited, with significant individual variability in responses (Widerström-Noga, 2023). These clinical features suggest that BLNP is not a static consequence of primary spinal pathology; its onset and persistence are underpinned by sustained pathological plasticity within the nociceptive system.

Existing mechanistic research on SCI-induced BLNP has mainly focused on intraspinal pathological changes, including local inflammation, glial responses, enhanced excitatory synaptic transmission, impaired inhibitory synaptic regulation, and dorsal horn neuronal hyperexcitability. These abnormalities can amplify residual sensory signals and facilitate their aberrant spinal transmission, providing an important substrate for central sensitization and neuropathic pain post-SCI (Kang et al., 2020). However, patients with similar degrees of injury may exhibit different pain phenotypes, and the distribution and intensity of pain may change over time. Therefore, mechanisms confined to spinal lesions cannot fully account for the persistence and clinical heterogeneity of BLNP. Central neuropathic pain also involves supraspinal neurons, glial cells, and immune signaling, as well as network-level changes among the thalamus, sensory cortex, insula, cingulate cortex, and brainstem modulatory structures (Rosner et al., 2023). Consequently, analysis of BLNP should consider aberrant sensory activity transmitted via residual ascending pathways and its subsequent processing in supraspinal structures, particularly the thalamus. Positioned between ascending spinal pathways and cortical pain-processing networks, the thalamus possesses distinct functional subdivisions. Lateral thalamic nuclei are primarily involved in discriminating the location, quality, and intensity of stimuli, whereas medial and intralaminar nuclei are more engaged in attention, affective experience, and behavioral responses. Bidirectional connections with the somatosensory cortex, insula, and anterior cingulate cortex enable the thalamus to both relay and regulate sensory information (Colloca et al., 2017). Following SCI, disruption of some spinothalamic fibers may reduce normal afferent input, while residual pathways may continue to transmit aberrant activity; molecular signals associated with injury may also alter the local thalamic environment. In this review, thalamic remodeling refers to sustained changes in neuroimmune signaling, neurotransmitter and metabolic states, intrinsic neuronal excitability and firing patterns, and connectivity with thalamus-related circuits following SCI. Human neuroimaging studies have provided clinically relevant evidence supporting such thalamic remodeling. Reports have documented structural, metabolic, and functional-connectivity abnormalities along nociceptive pathways in patients with SCI and neuropathic pain; however, deafferentation, motor impairment, and rehabilitation-related neuroplasticity can also reorganize the brain. Thus, identification of pain-related effects requires pain classification, preserved sensory function, and control groups with SCI but without neuropathic pain (Huynh et al., 2020). Although multiple SCI cohorts have reported bilateral thalamic gray-matter loss, this finding largely reflects the overall effects of SCI and does not establish a specific link with neuropathic pain on its own (Yu et al., 2022). Another systematic review on brain plasticity post-SCI similarly found that sensory deafferentation, functional recovery, and rehabilitation affect the structure and function of sensorimotor networks (Calderone et al., 2024). In contrast, a study including a group of SCI patients without neuropathic pain linked broader BLNP distribution to stronger functional connectivity between the posterior insula and lateral thalamic regions, providing more direct evidence for network differences associated with pain phenotypes (Huynh et al., 2021).

Previous reviews have typically discussed neuropathic pain from the perspective of spinal local central sensitization or widespread brain reorganization following SCI. A recent review on resting-state functional magnetic resonance imaging (MRI) summarized abnormalities in thalamocortical, insular, salience, limbic, sensorimotor, and descending modulatory networks (Ho et al., 2026). These findings still need to be integrated with evidence of local thalamic neuroimmune, neurochemical, and electrophysiological changes, and their applicability to BLNP remains to be elucidated. This article adopts a narrative review framework to sequentially discuss thalamic afferent states, neuroimmune signaling and neuron–glia communication, synaptic and ion-channel regulation, and the remodeling of related brain networks. Evidence is categorized into five groups according to its direct relevance to the present review, considering study populations or animal models, pain phenotypes, and outcome endpoints: (1) human studies clearly defined as BLNP; (2) human studies involving SCI-related neuropathic pain but with mixed or unspecified pain levels; (3) traumatic SCI animal studies that simultaneously assess nociceptive responses or hypersensitivity-related behaviors evoked by stimulation below the injury level and thalamic endpoints; (4) contextual SCI studies primarily focused on general SCI-related changes, at-level pain, or nonthalamic endpoints; and (5) indirect evidence from selective tract lesions, peripheral nerve injuries, basic physiology, or *in vitro* studies. These categories describe the direct relevance of evidence to this review’s topic rather than serving as a rating of study quality or causal strength. Local molecular or projection-specific interventions in animals can support the functional contribution of specific processes, while human imaging and electroencephalographic findings primarily provide clinical correlations. Integration across studies is used to construct a conceptual framework pending validation. Based on this, we evaluated the applicability of evidence in BLNP, unresolved controversies, and translational limitations to guide research on thalamus-related candidate biomarkers and mechanism-based interventions. Core animal studies are summarized in Table 1, human thalamic studies in Table 2, and contextual or indirect evidence in Supplementary Tables S1, S2.

**Table 1:** Core animal evidence for thalamic remodeling relevant to SCI neuropathic pain.

Study	Model and time point	Pain-related phenotype	Thalamic target and approach	Key thalamic finding	Interpretation and evidence boundary
Traumatic-SCI studies with below-level evoked hypersensitivity and a thalamic endpoint (Category 3)					
Zhao et al. (2007) Category 3	Male rats; T9 contusion; principal thalamic assessment at 4 weeks.	Mechanical and thermal evoked hypersensitivity.	VPL/ventrobasal thalamus; CCL21 assays, local CCL21 manipulation, microglial measures, and extracellular recording.	Thalamic CCL21 and VPL microglial reactivity increased. Intra-VPL CCL21 neutralization reduced microglial reactivity, evoked neuronal firing, and withdrawal hypersensitivity.	Strong local thalamic functional evidence. Spinal activity dependence was shown, but lesion-derived CCL21 transport to the thalamus and effects on spontaneous pain were not demonstrated.
Detloff et al. (2008) Category 3	Female rats; T8 contusion; remote spinal and thalamic tissue assessed from acute to chronic phases.	Below-level mechanical evoked hypersensitivity.	Whole-thalamus inflammatory assays; nucleus and cell type not resolved.	Animals with below-level hypersensitivity showed increased thalamic pro-inflammatory cytokines; remote lumbar inflammatory indices tracked hypersensitivity onset and severity.	Supports a remote inflammatory association, but lacks a nucleus-specific endpoint, local thalamic manipulation, and direct neuronal recording.
Wu et al. (2013) Category 3	Male rats; T10 spinal-segment contusion; 7 days and 8 weeks; systemic CR8 intervention.	Mechanical evoked hypersensitivity; greater PO activity in animals with marked hypersensitivity.	PO and VPL; Iba1 immunostaining, PO single-unit recording, cell-cycle assays, and CR8.	Microglial indices increased in PO/VPL and PO spontaneous and evoked firing increased. CR8 reduced immune, electrophysiological, and behavioral abnormalities.	Intervention-sensitive co-variation supports functional involvement, but systemic CR8 does not establish thalamus-specific mediation or a microglia-to-neuron causal sequence.
Tan et al. (2023) Category 3	Female rats; T9 clip-compression; tDCS over two 5-day blocks; assessment on day 12.	Thermal withdrawal response improved after tDCS.	VPL/thalamus; cytokines and CD86/Iba1 and CD206/Iba1 colabeling.	tDCS reduced TNF-α and IL-1β, increased IL-10, decreased CD86/Iba1, and increased CD206/Iba1 labeling.	tDCS was not thalamus-specific; immune and behavioral changes were concurrent. CD86/CD206 should not be interpreted as fixed, mutually exclusive microglial phenotypes.
Kubota et al. (2026) Category 3	Male mice; T9-T10 contusion; 4 days to 12 weeks.	Below-level mechanical and thermal withdrawal outcomes.	Whole thalamus; CD11b immunostaining and p38/ERK1/2 colocalization.	Thalamic CD11b-positive cell number was unchanged, whereas CD11b colocalization with phosphorylated p38 or ERK1/2 increased at selected acute and chronic time points.	Shows pathway- and phase-dependent immune signaling; nucleus specificity and a pain-specific thalamic link were not established.
Hains et al. (2005) Category 3	Male rats; T9 contusion; chronic assessment at 4 weeks.	Mechanical and thermal evoked hypersensitivity with enlarged receptive fields.	VPL recording; VPL/VPM Nav1.3 expression; lumbar intrathecal Nav1.3 antisense.	Nav1.3 expression and VPL spontaneous/evoked activity, afterdischarge, and receptive-field size increased; antisense reduced these abnormalities.	Supports Nav1.3-related hyperresponsiveness, but lumbar delivery does not isolate a thalamic Nav1.3 mechanism.
Gwak et al. (2010) Category 3	Male rats; unilateral T13 hemisection; 4 weeks.	Bilateral hindpaw mechanical evoked hypersensitivity.	Bilateral VPL extracellular recording during peripheral stimulation.	Stimulus-evoked responses increased in both VPLs after unilateral injury, together with bilateral behavioral hypersensitivity.	Demonstrates bilateral VPL response enhancement, but co-occurrence does not establish thalamic causality or spontaneous pain.
He et al. (2025) Category 3	Female rats; T9 vertebral laminectomy followed by contusion; chronic assessment at 6 weeks.	Below-level mechanical or thermal evoked hypersensitivity.	c-Fos mapping in medial thalamus, dorsal horn, locus coeruleus, and S1 after noxious stimulation.	Noxious stimulation recruited medial thalamic, deep dorsal-horn, and locus-coeruleus neurons without a corresponding increase in S1.	Provides regional activity mapping; c-Fos is an indirect, temporally limited measure and does not test thalamic necessity.
Fukushi et al. (2026) Category 3	Male rats; T9-T10 contusion; intravenous MSCs on day 3; assessment at day 28.	Below-level mechanical withdrawal hypersensitivity improved after MSC treatment.	Intralaminar thalamic nuclei; dendritic-spine and gene-expression analyses.	SCI increased mature mushroom-spine density and cytoskeletal/AMPA-regulatory transcripts; MSC treatment attenuated structural, molecular, and behavioral changes.	Extends evidence to postsynaptic structure, but systemic MSC treatment does not establish thalamic mediation.
Liu et al. (2024) Category 3	Mice; moderate contusion SCI; chronic phase.	Chronic mechanical evoked hypersensitivity; innocuous touch recruited lateral-thalamic and cortical neurons.	Lateral-thalamic TRAP2 ensembles; tracing and chemogenetic/corticospinal-neuron manipulations.	Inhibiting touch-responsive lateral-thalamic ensembles or relevant corticospinal neurons reduced hypersensitivity and cortical activation; thalamic collaterals increased after SCI.	Supports within-model neuronal and circuit involvement; interventions were not uniformly projection-terminal specific and did not assess spontaneous human BLNP.
Zhao et al. (2024) Category 3	Female mice; T10 contusion; M1 high-frequency rTMS beginning 1 or 2 weeks after SCI.	Mechanical, cold, and heat evoked hypersensitivity improved after rTMS.	Motor cortex-thalamus connectivity; thalamic/hypothalamic transcriptomics; spinal inflammatory markers.	rTMS strengthened motor cortex-thalamus connectivity and altered transcriptomic and spinal inflammatory measures alongside behavioral improvement.	The intervention was not thalamus-specific; the relative contributions of cortical-thalamic, transcriptomic, and spinal changes remain unresolved.
Selected indirect mechanistic evidence from non-traumatic spinothalamic-tract lesions (Category 5)					
Wang and Thompson (2008) Category 5	Male rats; unilateral electrolytic or demyelinating T8/T9 STT lesions; main slice assessment at day 14.	Persistent forepaw and hindpaw mechanical/thermal withdrawal hypersensitivity.	VPL slice field potentials; ethosuximide *in vitro* and systemic behavioral testing.	VPL population bursting increased; ethosuximide suppressed bursting in vitro and reduced hypersensitivity systemically.	Indirect, non-traumatic lesion evidence. Systemic drug effects do not establish VPL-specific behavioral mediation.
Park et al. (2018) Category 5	*Gad2*-Cre mice; unilateral C7 electrolytic STT lesion; recordings at 21–35 days.	Mechanical and thermal withdrawal hypersensitivity after selective STT lesion.	PO slice whole-cell recording of GABAA- and GABAB-receptor-mediated currents.	Tonic presynaptic GABAB regulation at GABAergic terminals was lost and GABAB inhibition of glutamate release was weakened; tonic extrasynaptic GABAA current was unchanged.	Defines a PO inhibitory mechanism in a selective tract-lesion model; traumatic-SCI replication and *in vivo* behavioral rescue are lacking.
Cha et al. (2013) Category 5	Male rats; unilateral C7 electrolytic STT lesion; electrophysiology at 14 days or later.	Withdrawal hypersensitivity after selective STT lesion.	M1 stimulation, ZI muscimol, and ZI/PO single-unit recording.	M1 stimulation activated a subset of ZI neurons and suppressed many PO neurons; ZI inactivation abolished most PO inhibitory responses.	Supports an M1-ZI-PO inhibitory pathway, but the lesion and interventions were not equivalent to traumatic SCI or modern projection-specific manipulation.

Evidence categories indicate relevance to the review question, not study quality or causal certainty. Category 3 comprises traumatic-SCI studies with below-level evoked hypersensitivity and a thalamic endpoint; Category 5 denotes indirect evidence. Evoked withdrawal or hypersensitivity outcomes are not equivalent to spontaneous clinical BLNP.

AMPA, alpha-amino-3-hydroxy-5-methyl-4-isoxazolepropionic acid; BLNP, below-level neuropathic pain; CCL21, C-C motif chemokine ligand 21; ERK, extracellular signal-regulated kinase; GABA, gamma-aminobutyric acid; GABAA/GABAB, GABA type A/type B receptor; Iba1, ionized calcium-binding adaptor molecule 1; IL, interleukin; M1, primary motor cortex; MSC, mesenchymal stem cell; PO, posterior thalamic nucleus; rTMS, repetitive transcranial magnetic stimulation; S1, primary somatosensory cortex; SCI, spinal cord injury; STT, spinothalamic tract; tDCS, transcranial direct current stimulation; TNF, tumor necrosis factor; TRAP2, second-generation targeted recombination in active populations; VPL, ventral posterolateral nucleus; VPM, ventral posteromedial nucleus; ZI, zona incerta.

**Table 2:** Human thalamic evidence in SCI-related neuropathic pain.

Study	Cohort and pain phenotype	Modality and thalamic target	Main finding	Interpretation and evidence boundary
Pain-phenotype-related thalamic structural, metabolic, perfusion, and pathway evidence (Categories 1–2)				
Pattany et al. (2002) Category 2	SCI with chronic neuropathic pain *n* = 7; pain-free SCI *n* = 9; healthy controls *n* = 10. Pain level was not classified as at- versus below-level.	Bilateral thalamic proton MRS.	N-acetyl compounds were lower in the pain group and inversely related to pain intensity; myo-inositol was positively related to pain intensity.	Supports regional metabolic association. Small cross-sectional cohort, older pain classification, and no nucleus or cell-type resolution.
Gustin et al. (2014) Category 1	Complete thoracic SCI with persistent BLNP *n* = 12; injury-matched pain-free SCI *n* = 11; healthy controls *n* = 21.	Thalamic proton MRS and ASL perfusion, including the TRN region.	The BLNP group had lower thalamic NAA/Cr and GABA/Cr and lower perfusion in the TRN region.	Relatively direct human BLNP association because an injury-matched pain-free SCI group was included; still cross-sectional and region-level.
Widerström-Noga et al. (2015) Category 2	Chronic SCI with high pain impact *n* = 19, low pain impact *n* = 35, and able-bodied controls *n* = 24; pain groups were not defined solely by level.	Thalamic proton MRS combined with quantitative sensory testing.	Greater pain intensity was associated with lower thalamic Glx/Ins and NAA/Ins ratios and relatively preserved below-level sensory function.	Links sensory preservation, pain intensity, and regional metabolites; correlational design and unresolved nuclear/cellular specificity.
Kyathanahally et al. (2021) Category 2	Chronic traumatic SCI with neuropathic pain *n* = 13; pain-free SCI *n* = 17; healthy controls *n* = 23. Pain could be at or below level.	Multiparametric quantitative MRI; lateral and medial thalamic R2* and volume.	Pain-related R2* differences were mainly lateral, whereas SCI-related thalamic volume loss was mainly medial.	Separates pain-associated tissue metrics from general SCI-related atrophy; R2* is indirect and pain subtype was mixed.
Dong et al. (2023) Category 2	Chronic SCI with neuropathic pain *n* = 30 and pain-free SCI *n* = 15; at-level, below-level, and combined pain phenotypes were included.	Diffusion MRI of posterior thalamic radiation and distributed white-matter tracts.	Pain-related diffusion differences involved posterior thalamic radiation, corpus callosum, internal capsule, corona radiata, cingulum, and association fibers.	Thalamus-related pathway evidence rather than a nucleus-specific thalamic measure; diffusion metrics should not be equated with tract function.
Gustin et al. (2023) Category 1	Complete thoracic SCI with persistent BLNP *n* = 7; uncontrolled 10-day immersive virtual-walking intervention.	Pre-post thalamic proton MRS.	Pain ratings decreased and thalamic GABA/Cr increased, but the magnitudes of change were not significantly correlated.	Shows modifiability of a candidate thalamic signal; very small uncontrolled study and no evidence that GABA change mediated pain improvement.
Pain-related thalamic functional-connectivity evidence (Category 2)				
Huynh et al. (2021) Category 2	Thoracic SCI with neuropathic pain *n* = 19, pain-free SCI *n* = 10, and healthy controls *n* = 25; all pain participants had below-level pain, and 9 also had at-level pain.	Structural MRI and resting-state fMRI; posterior insula and lateral/mediodorsal/ventroposterior thalamic regions.	Overall neuropathic-pain extent correlated with stronger posterior-insula-thalamic connectivity; thalamic volume was also related to SCI and pain measures.	Clinically relevant thalamic association, but the analysis did not isolate a BLNP-only effect and cannot establish direction or cellular mechanism.
Mandloi et al. (2023, 2024); Shoraka et al. (2024)^a^ Category 2	Small chronic-SCI cohort series: moderate/severe neuropathic pain *n* = 7, none/mild pain *n* = 3, and healthy controls *n* = 10; pain level was not BLNP-specific.	Resting-state fMRI; insula/ACC, PAG-thalamic connections, and thalamic subdivisions.	The series reported insula-frontal/ACC connectivity changes and nucleus-dependent differences in PAG-thalamic and thalamic-network connectivity.	Exploratory, cross-sectional evidence with very small groups. Reports are treated as a cohort series rather than independent replication.
General SCI-related thalamic remodeling without pain-specific analysis (Category 4)				
Karunakaran et al. (2020) Category 4	Complete thoracolumbar SCI *n* = 19 and healthy controls *n* = 22; pain status was not recorded.	Resting-state connectivity of mediodorsal, ventrolateral, and pulvinar thalamic subdivisions.	SCI was associated with reduced mediodorsal/ventrolateral connectivity and increased pulvinar-frontal/parietal connectivity.	Establishes general SCI-related thalamocortical reorganization but cannot be interpreted as pain-specific evidence.
Yu et al. (2022) Category 4	Voxel-based morphometry meta-analysis of 20 studies: SCI *n* = 410 and healthy controls *n* = 406; pain stratification was not primary.	Whole-brain structural MRI meta-analysis; bilateral thalamus.	SCI was associated with bilateral thalamic gray-matter volume reduction.	Supports general structural remodeling after SCI; it does not establish an association with neuropathic pain.

Category 1 denotes an explicitly defined BLNP phenotype; Category 2 denotes mixed or incompletely classified SCI-related neuropathic pain; Category 4 denotes general SCI-related remodeling without pain-specific analysis. Imaging and connectivity findings are associative candidate clinical measures, not validated biomarkers.

^a^The three reports used the same group sizes and highly similar cohort descriptions and are therefore presented as a cohort series rather than independent replication.

ACC, anterior cingulate cortex; ASL, arterial spin labeling; BLNP, below-level neuropathic pain; Cr, creatine; fMRI, functional magnetic resonance imaging; GABA, gamma-aminobutyric acid; Glx, glutamate plus glutamine; Ins, myo-inositol; MRS, magnetic resonance spectroscopy; NAA, N-acetylaspartate; NP, neuropathic pain; PAG, periaqueductal gray; R2*, effective transverse relaxation rate; SCI, spinal cord injury; TRN, thalamic reticular nucleus.

## 2 Afferent substrates and initial conditions for thalamic remodeling after SCI

SCI disrupts ascending spinal pathways and impairs signal conduction in residual fibers, thereby altering the intensity, sensory modality, and temporal patterns of thalamic afferent inputs. The spinothalamic system comprises functionally distinct projections. The ventral posterolateral nucleus (VPL) receives somatotopically organized inputs and is primarily involved in discriminating stimulus location, quality, and intensity. The posterior thalamic nuclei (PO) integrate ascending spinal signals with cortical inputs, while the medial and intralaminar nuclei are more closely involved in arousal, attention, and affective-motivational responses associated with pain (Colloca et al., 2017). Due to differences in afferent sources and cortical projections, thalamic responses after SCI vary by nucleus, laterality, and time since injury. Approximately 30 days after thoracic contusion, responses to somatic and visceral stimuli below the injury level changed in multiple thalamic regions. New responses emerged during cutaneous tactile stimulation at the injury level, predominantly in ventral and posterior thalamic regions, and were more common in animals exhibiting at-level touch-evoked pain-like behavior (Hubscher and Johnson, 2006). Unilateral SCI led to increased bilateral stimulus-evoked firing in VPL neurons, accompanied by bilateral hindlimb hypersensitivity (Gwak et al., 2010). Continuous recordings after hemisection of the thoracic spinal cord showed early changes in VPL stimulus responses, followed by partial recovery over subsequent weeks (Liang and Mendell, 2013). Thus, SCI does not uniformly increase activity in all thalamic nuclei; the distribution, laterality, and temporal course of sensory responses depend on the injury model and stage of injury.

Whether sensory activity generated below the injury level can still reach the thalamus depends on the status of spared ascending pathways. Clinically complete SCI is a functional classification based on neurological examination and does not equate to complete anatomical transection of the spinal cord. In a study of 11 patients with chronic complete SCI, tactile or nociceptive stimulation in areas without sensation below the injury level elicited activity in the primary somatosensory cortex corresponding to the stimulated site in six subjects, suggesting that some patients retained somatosensory connections undetected by routine examinations (Awad et al., 2020). A longitudinal MRI study further found that the width of ventral tissue bridges early after SCI was associated with subsequent neuropathic pain, pain intensity, and pinprick sensory scores, supporting the coexistence of spared ascending conduction and pain from the perspective of residual spinal structures (Pfyffer et al., 2020). Wydenkeller et al. used contact heat evoked potentials (CHEPs) to assess the function of the spinothalamic pathway. CHEPs were abnormal in 94% of BLNP patients compared with 71% of non-neuropathic pain SCI patients, with no statistically significant difference between the two groups. After adjusting for deafferentation, the peak frequency within the 6–12 Hz band remained lower in the pain group in resting electroencephalography (EEG) (Wydenkeller et al., 2009). These human studies provide complementary evidence regarding spared structures, cortical responses, and conduction, indicating that there is no simple linear relationship between residual ascending pathways and pain phenotypes. Partial damage to the spinothalamic tract can produce both sensory deafferentation and aberrant residual conduction. Sensory deafferentation describes reduced normal patterned input to the thalamus resulting from partial fiber interruption, rather than a complete loss of ascending conduction. Aberrant residual conduction refers to the persistent transmission of spontaneous, stimulus-evoked, or temporally distorted neural activity via spared fibers. In models of selective electrolytic or demyelinating spinothalamic tract lesions, thalamic neurons exhibited inhibitory synaptic regulation and altered intrinsic membrane properties, accompanied by persistent hyperexcitability (Wang and Thompson, 2008). Therefore, sensory deafferentation may alter the state of thalamic processing of afferent signals, while spared pathways provide a route for aberrant activity to reach the thalamus. Molecular signals associated with SCI may also affect distant thalamic regions. Animal studies have shown that injured or stressed neurons can release CCL21 (de Jong et al., 2005), and thalamic CCL21 levels change with spinal conduction activity (Zhao et al., 2007). Reduced normal afferent input, aberrant activity in spared pathways, and injury-related molecular signals modify different aspects of thalamic afferents and the local environment, thereby creating conditions for subsequent neuroimmune and neuron–glia responses.

## 3 Thalamic Neuroimmune remodeling and regulation of neuronal responsiveness

Thalamic neuroimmune changes after SCI primarily include microglial responses and changes in chemokine and cytokine expression, with their spatial distribution and temporal progression varying across thalamic subnuclei and injury stages. Simultaneous measurements of glial markers, neuronal activity, and pain-related behaviors in both the VPL and PO help elucidate the relationship between neuroimmune status and thalamic information processing.

### 3.1 Nuclear distribution and temporal evolution of microglial responses

In rats with thoracic SCI, VPL microglia gradually exhibit reactive morphological changes, including somatic hypertrophy, process retraction, and reduced branching. Cluster of differentiation 11b/c (CD11b/c) immunoreactivity begins to increase 2 weeks post-injury and further rises by 4 weeks; mechanical paw-withdrawal thresholds decrease during the same period (Zhao et al., 2007). Thus, VPL microglial responses develop gradually over several weeks post-injury, following a delayed temporal course that parallels mechanical hypersensitivity-related behaviors. In addition to these temporal changes, thalamic microglial responses differ across nuclei and sides after SCI. At 7 days and 8 weeks post-spinal contusion, the proportion of ionized calcium-binding adaptor molecule 1 (Iba1)-positive cells and immunoreactivity increased in both PO and VPL. The adjacent centrolateral nucleus showed no significant changes. The ventral posteromedial nucleus (VPM) exhibited increased Iba1 immunoreactivity only at 8 weeks, while the proportion of Iba1-positive cells remained unchanged. At the same time points, PO neurons displayed increased spontaneous firing and sensory stimulus-evoked firing, with stronger neuronal activity observed in animals exhibiting more pronounced mechanical hypersensitivity (Wu et al., 2013). In another study, using unilateral electrolytic spinothalamic tract lesions in rats, Iba1 immunoreactivity increased in the ipsilateral VPL on days 21 and 28 and in the contralateral VPL on day 28, accompanied by bilateral mechanical hypersensitivity-related behaviors. Estradiol treatment attenuated VPL microglial responses and pain-related behaviors (Saghaei et al., 2013). These two studies demonstrate nucleus-dependent differences and bilateral temporal patterns, indicating that thalamic microglial responses after SCI are neither immediate nor uniform. Studies examining multiple nervous-system regions provide additional spatiotemporal context for distal responses. In mice with traumatic SCI, CD11b-positive microglia/macrophages peaked at day 14 at the injury site and increased in the lumbar enlargement during the chronic phase; CD11b-positive cells also increased in the hippocampus and amygdala by week 12. The number of CD11b-positive cells in the thalamus did not change significantly, but colocalization signals of CD11b with phosphorylated p38 mitogen-activated protein kinase or extracellular signal-regulated kinase 1/2 (ERK1/2) increased during the acute phase and at week 12 (Kubota et al., 2026). A systematic review of 20 animal studies similarly found that increased microglial density or activity after SCI may involve distal spinal regions and specific brain areas (Ramos and Cruz, 2023). These findings demonstrate regional and phase-dependent differences in distal neuroimmune changes; evidence linking the thalamus more directly to below-level sensory hypersensitivity remains primarily focused on VPL and PO.

### 3.2 CCL21-mediated neuron–microglia signaling

CCL21 is an important chemokine mediating signaling from injured neurons to microglia. Cell biology studies have demonstrated that glutamate stimulation upregulates the expression and release of CCL21 in cultured cortical neurons. CCL21 synthesized by neurons can be packaged into secretory vesicles, transported along neurites to presynaptic structures, and induce microglial migration upon release (de Jong et al., 2005). Live-cell imaging further confirmed that neuron-derived CCL21 is primarily sorted into large dense-core vesicles and preferentially transported along axons to nerve terminals, providing a structural basis for CCL21 release at sites distant from the neuronal soma (de Jong et al., 2008). The regulatory role of CCL21 on microglial function was first demonstrated in peripheral nerve injury models. Following L5 spinal nerve transection in mice, injured primary sensory neurons rapidly express CCL21 and transport it to central terminals in the spinal dorsal horn. CCL21 promotes the expression of P2X4 purinergic receptors in spinal microglia. CCL21-deficient mice do not exhibit corresponding P2X4 receptor upregulation or tactile allodynia-related behaviors, and intrathecal administration of CCL21-blocking antibodies also attenuates these behaviors in wild-type mice (Biber et al., 2011). These studies establish the biological basis for CCL21 as a neuron-derived signal regulating microglial function, and further experimental support has been obtained for its role in distal thalamic neuroimmune responses after SCI.

Using an adult male rat T9 contusion model, Zhao et al. found enhanced CCL21 labeling in lumbosacral dorsal horn neurons 4 weeks post-injury, alongside elevated CCL21 levels in T9 and L4 dorsal horn tissue and thalamic VPL. The researchers subsequently tested whether the increase in thalamic CCL21 depends on ascending spinal activity. In rats without SCI, unilateral electrical stimulation of the spinothalamic region in the ventrolateral funiculus at L1 increased CCL21 levels in the ipsilateral ventrobasal thalamic complex. In rats with SCI, lidocaine blockade followed by complete transection at T1 or L1 reduced thalamic CCL21 levels, with a greater reduction observed after T1 intervention (Zhao et al., 2007). These results indicate that changes in thalamic CCL21 levels after SCI depend at least partially on activity conducted through the spinal cord. The same study evaluated local effects by supplementing or neutralizing CCL21 within the VPL. Intraventricular injection of recombinant CCL21 into the VPL of uninjured rats produced a transient microglial response, decreased mechanical withdrawal thresholds, and shortened thermal withdrawal latencies; coadministration of minocycline attenuated these effects. In rats with SCI, intra-VPL injection of a CCL21-neutralizing antibody reduced the microglial response relative to a nonspecific immunoglobulin G (IgG) control. Neutralization also reduced wide dynamic range neuron-evoked firing during brushing, pressure, and pinch of the hind paw, without significantly altering spontaneous activity under unstimulated conditions; mechanical withdrawal thresholds increased, and thermal withdrawal latencies lengthened (Zhao et al., 2007). These findings support a role for VPL-derived CCL21 in neuron–microglia signaling after SCI, as well as its association with modulated neuronal stimulus-responsiveness and hypersensitivity-related behaviors.

### 3.3 Inflammation-related signaling and thalamic neuronal responsiveness

A study combining systemic cell-cycle inhibition, PO recordings and *in vitro* experiments investigated the relationship between inflammation-related signaling and thalamic neuronal reactivity. Following thoracic spinal cord contusion in rats, PO neurons exhibited increased spontaneous firing rates and enhanced evoked firing in response to peripheral sensory stimuli; rats exhibiting significant mechanical hypersensitivity-related behavior showed higher spontaneous firing rates in the PO. Intraperitoneal administration of the cyclin-dependent kinase inhibitor CR8 for 7 days, beginning 3 h post-injury, attenuated chronic-phase mechanical hypersensitivity-related behavior. CR8 treatment also reduced spontaneous and stimulus-evoked firing in the PO, as well as the expression of cell-cycle-related proteins and microglial reactivity. *In vitro* experiments demonstrated that treating primary neurons with conditioned media from lipopolysaccharide-stimulated microglia increased neuronal expression of cyclin D1 and CCL21. Pretreatment of microglia with CR8 attenuated the conditioned media-induced changes in neuronal molecular expression (Wu et al., 2013). These findings support a functional relationship between cell-cycle-related processes, soluble mediators released by microglia, and molecular and electrophysiological changes in PO neurons.

Changes in proinflammatory cytokines reflect the extension of neuroimmune responses to distal sites and their temporal evolution following SCI. After moderate thoracic SCI in rats, microglial reactivity in the L5 dorsal horn, distant from the injury site, was enhanced and correlated with decreased mechanical withdrawal thresholds in the hind paw. Tumor necrosis factor-α (TNF-α) and interleukin-1β (IL-1β) in the L5 dorsal horn increased primarily on days 7 and 21 post-injury, while interleukin-6 (IL-6) increased on days 21 and 35. The same study found that rats exhibiting below-level mechanical hypersensitivity-related behavior also had elevated levels of proinflammatory cytokines in the thalamus (Detloff et al., 2008). Another animal study demonstrated that 1 month after SCI, there was enhanced activation of the Janus kinase 2 (JAK2)/signal transducer and activator of transcription 3 (STAT3) signaling pathway in L4–L5 dorsal horn microglia and astrocytes, accompanied by increased dorsal horn neuronal reactivity. JAK2 inhibitors attenuated glial responses and mechanical and thermal hypersensitivity-related behavior, while IL-6 neutralizing antibodies reduced JAK2/STAT3 signaling and pain-related behaviors. Minocycline treatment in the same study was also associated with reduced dorsal horn neuronal hyperresponsiveness (Lee et al., 2023). Detloff et al. demonstrated inflammatory changes in both the distal lumbar spinal cord and thalamus, with phase-dependent changes observed in the lumbar cord, whereas Lee et al. provided molecular evidence for lumbar glia–neuron regulation. Transcranial direct current stimulation (tDCS) further indicated that thalamic inflammatory markers can change with intervention. Following two 5-day courses of tDCS, thermal paw-withdrawal latencies were prolonged in spinal cord-injured rats, and thalamic TNF-α and IL-1β levels were reduced, while interleukin-10 (IL-10) levels increased. The proportion of cluster of differentiation 86 (CD86)/Iba1-double-positive cells in the VPL decreased, while that of cluster of differentiation 206 (CD206)/Iba1-double-positive cells increased; total Iba1 protein and reactive microglial morphology did not recover in parallel (Tan et al., 2023). These results indicate that tDCS altered thalamic inflammatory mediator levels and the VPL microglial marker-expression profile, accompanied by improved responses to thermal stimulation. Since CD86 and CD206 are not mutually exclusive markers of fixed phenotypes, these changes should be interpreted in conjunction with Iba1 expression and cellular morphology.

Thalamic neuroimmune responses following SCI also vary by cell type, brain region, and observation time. Following unilateral electrolytic spinothalamic tract injury in rats, Iba1 immunoreactivity in the VPL gradually increased over several weeks, while glial fibrillary acidic protein (GFAP) expression did not show a corresponding increase (Saghaei et al., 2013). At 24 h after mild T10 contusion in female rats, thalamic GFAP transcript and protein levels were reduced, while thalamic Iba1 transcript and protein levels remained unchanged (Mandwie et al., 2022). These results indicate that glial markers in the thalamus follow distinct trajectories following SCI. Inflammation-related changes also extend to supraspinal regions involved in pain processing. In female mice with SCI, acute reflexive hypersensitivity was characterized by a transient increase in spinal inflammatory factors, whereas chronic affective, nonreflexive pain-related behaviors were associated with glial responses in the medial prefrontal cortex and enhanced signaling involving C-X3-C motif chemokine ligand 1 (CX3CL1) and its receptor, C-X3-C motif chemokine receptor 1 (CX3CR1) (Castany et al., 2023). A systematic review of nine animal studies summarized changes in microglia, astrocytes, cytokines, and chemokines in the thalamus, prefrontal and cingulate cortices, amygdala, and brainstem modulatory structures following SCI (Ferreira et al., 2026). These studies place thalamic neuroimmune changes within a distributed supraspinal inflammatory response framework. Experimental evidence directly linking local thalamic inflammatory signals to neuronal firing and below-level hypersensitivity-related behavior remains concentrated in the VPL and PO. Overall, thalamic microglial responses, along with changes in CCL21, cell-cycle-related proteins, and proinflammatory cytokines, may coexist with increased spontaneous or stimulus-evoked firing, suggesting that local neuroimmune status may modulate thalamic neuronal responsiveness.

## 4 Thalamic neurochemical and electrophysiological remodeling

Thalamic remodeling following SCI also involves changes in synaptic regulation, regional metabolism and perfusion, ion channels, and firing patterns. Animal studies can examine neurotransmitter and electrophysiological mechanisms within specific nuclei, while human proton magnetic resonance spectroscopy (^1^H-MRS) and arterial spin labeling (ASL) reflect metabolite composition and perfusion in specific voxels or regions, respectively. While these techniques target distinct biological scales, their combined application enables comprehensive profiling of multilevel alterations in thalamic neurochemistry, synaptic processing and neuronal firing after SCI.

### 4.1 Excitatory-inhibitory synaptic regulation and synaptic structural changes

The spinothalamic tract and corticothalamic projections provide major glutamatergic input to thalamic relay neurons. Projections releasing gamma-aminobutyric acid (GABA) from the thalamic reticular nucleus (TRN), zona incerta (ZI), and other inhibitory sources regulate the magnitude and timing of relay-neuron responses. In rats with unilateral electrolytic lesions of the spinothalamic tract, *in vivo* microdialysis combined with high-performance liquid chromatography showed that extracellular glutamate levels in the ipsilateral VPL were elevated on days 14 and 28 post-injury, accompanied by mechanical hypersensitivity- and thermal hyperalgesia-related behaviors. Administration of 17β-estradiol after injury reduced VPL extracellular glutamate levels and attenuated these behavioral abnormalities (Naderi et al., 2014). Extracellular glutamate levels were determined jointly by release, uptake, and metabolic conversion. This study demonstrated that VPL glutamate homeostasis was altered following spinothalamic tract injury, accompanied by hypersensitivity-related behaviors, but did not distinguish the specific processes leading to increased glutamate.

Whole-cell patch-clamp recordings of brain slices elucidated the inhibitory synaptic regulation mechanisms in the PO. Under normal conditions, PO neurons receive synaptic GABA_A_ receptor-mediated inhibition, and some neurons also exhibit tonic inhibitory currents mediated by extrasynaptic GABA_A_ receptors. Presynaptic GABA_B_ receptors on GABAergic and glutamatergic terminals regulate the release of GABA and glutamate, respectively (Park et al., 2017). In mice with a unilateral C7 electrolytic spinothalamic-tract lesion and hypersensitivity-related behavior, tonic GABA_B_ receptor-mediated regulation at GABAergic terminals was lost, and GABA_B_ receptor-mediated inhibition of glutamate release was significantly reduced; tonic inhibitory currents mediated by extrasynaptic GABA_A_ receptors did not change significantly (Park et al., 2018). These findings indicate that changes in inhibitory regulation in the PO after SCI primarily involve impaired function of presynaptic GABA_B_ receptors, which can alter the regulation of GABA and glutamate release as well as affect the integration of ascending spinal inputs and cortical feedback in the PO. The reproducibility of these findings in traumatic SCI remains to be established. Changes in synaptic function may also be accompanied by postsynaptic structural remodeling. A recent comparative study between peripheral nerve injury and SCI models found that SCI preferentially increased the density of mature mushroom-shaped dendritic spines in intralaminar thalamic nuclei and upregulated *Akap5* (A-kinase anchoring protein 5), *Actr2* (actin-related protein 2), and *Sorbs2* (sorbin and SH3 domain containing 2), genes involved in actin cytoskeletal organization and α-amino-3-hydroxy-5-methyl-4-isoxazolepropionic acid (AMPA) receptor regulation. Intravenous administration of mesenchymal stem cells attenuated changes in dendritic spines and gene expression, accompanied by improvements in pain-related behaviors (Fukushi et al., 2026). This study extends thalamic abnormalities after SCI from neurotransmitter and receptor regulation to postsynaptic structure, suggesting that different types of nervous-system injury may affect different thalamic regions.

### 4.2 Human evidence of changes in thalamic metabolites, GABA, and perfusion

^1^H-MRS detects *in vivo* neurochemical signals within defined voxels, providing human evidence for analyzing the biochemical state of the thalamus related to pain after SCI. An early study included 7 participants with paraplegia and chronic neuropathic pain, 9 pain-free participants with paraplegia, and 10 healthy controls. Thalamic N-acetyl-containing compound concentrations were negatively correlated with pain intensity, while myo-inositol (Ins) concentrations were positively correlated; N-acetyl-containing compound concentrations were lower in the pain group than in the pain-free group (Pattany et al., 2002). The signal for N-acetyl-containing compounds is primarily composed of N-acetylaspartate (NAA), which is commonly used to characterize neuronal metabolic status. Ins participates in cellular osmoregulation and plays an important role in glial-related metabolism. These findings suggest that chronic neuropathic pain is associated with alterations in the composition of thalamic neuronal- and glial-associated metabolites. Another study involving participants with complete thoracic SCI found that 12 participants with BLNP had lower thalamic NAA/Cr and GABA/Cr ratios compared with 11 participants with similar injury characteristics but without neuropathic pain; Cr denotes the creatine-related spectroscopic signal. ASL also showed reduced cerebral blood flow in the region of the TRN (Gustin et al., 2014). Using pain-free SCI controls with similar injury characteristics helps identify pain-associated thalamic changes. NAA/Cr, GABA/Cr, and regional perfusion reflect neuronal metabolism, GABA-related neurochemical signals, and tissue blood-flow status, respectively. These metrics provide evidence of biochemical and functional state changes in the thalamus in BLNP across different levels, but should not be treated as interchangeable readouts for a single physiological process.

A study combining ^1^H-MRS with quantitative sensory testing further found that participants with SCI and higher pain intensity had lower thalamic ratios of combined glutamate and glutamine signal (Glx) to Ins and NAA to Ins, while retaining relatively more thermal and vibratory sensory function below the injury level (Widerström-Noga et al., 2015). This finding indicates an association between the degree of preserved below-level sensory function, pain intensity, and thalamic metabolite composition. Glx/Ins reflects the combined glutamate-plus-glutamine signal relative to myo-inositol within the same voxel and can be used to describe the neuronal-glial metabolic status of thalamic regions. A preliminary intervention study suggested that thalamic GABA-related signals may change over time. After completing 10 days of immersive interactive virtual walking training, 7 participants with complete thoracic SCI and persistent BLNP showed an average reduction in pain scores and an increase in thalamic GABA/Cr, although the magnitude of these two changes was not significantly correlated (Gustin et al., 2023). Thus, sensorimotor training may be accompanied by changes in thalamic GABA-related signals, although controlled studies are still needed to clarify the causal relationship with pain improvement. Overall, human imaging studies link neuropathic pain after SCI to regional changes in thalamic NAA, Ins, Glx, GABA, and perfusion, delineating candidate thalamic neurochemical phenotypes rather than directly measuring synaptic neurotransmission.

### 4.3 Ion-Channel changes, abnormal firing, and enhanced stimulus responsiveness

Voltage-gated ion channels regulate the magnitude and temporal patterns of thalamic responses to synaptic input by shaping action-potential thresholds, repolarization, and repetitive firing. Four weeks after T9 contusion in rats, expression of the voltage-gated sodium channel Na_v_1.3 was increased in neurons of the VPL and VPM. Extracellular single-unit recordings from the VPL showed increased spontaneous firing, enhanced responses to both innocuous and noxious peripheral stimuli, prolonged after-discharges, and expanded peripheral receptive fields. Following complete transection at the rostral end of the original injury site, some abnormalities persisted, indicating that chronic thalamic hyperresponsiveness was not entirely dependent on continuing ascending spinal activity. Intrathecal lumbar administration of Na_v_1.3 antisense oligonucleotides reduced Na_v_1.3 expression in thalamic neurons and attenuated electrophysiological abnormalities (Hains et al., 2005). Na_v_1.3 expression was also associated with the temporal organization of burst firing. After SCI, VPL neurons generated fewer action potentials per burst, bursts were shorter in duration, and intra-burst and inter-burst intervals were prolonged; antisense treatment for Na_v_1.3 partially restored these parameters (Hains et al., 2006). Thus, aberrant Na_v_1.3 expression not only affects overall excitability but also influences the temporal organization of action potentials within bursts, potentially having downstream consequences for thalamic output coding.

Different thalamic electrophysiological abnormalities exhibit distinct relationships with the hypersensitivity phenotype. At 7 days after contusion, the proportion of VPL neurons exhibiting oscillatory spontaneous firing increased regardless of whether mechanical allodynia-related behaviors are present. In contrast, enhanced whisker responses and prolonged post-stimulus after-discharges occur primarily in animals displaying mechanical allodynia-related behaviors (Gerke et al., 2003). Therefore, oscillatory spontaneous activity may reflect relatively widespread electrophysiological plasticity following SCI, whereas enhanced stimulus-evoked responses and after-discharges are more closely linked to the hypersensitivity phenotype. Activity-dependent markers have extended single-unit findings to neuronal populations. Following persistent pain-related behaviors induced by C5–C6 contusion in mice, labeling of dorsal horn projection neurons and several classes of excitatory interneurons increased; labeled neurons increased in the thalamus but decreased in the midbrain periaqueductal gray (PAG) (Brown et al., 2022). In rats exhibiting below-level hypersensitivity after thoracic contusion, noxious mechanical or thermal stimulation increased the number of c-Fos-positive neurons in the deep dorsal horn, medial thalamus, and locus coeruleus, without corresponding increases in the primary somatosensory cortex (He et al., 2025). These population-level studies map the regional recruitment of abnormal activity, while firing rates and timing require direct single-neuron recordings.

Existing evidence identifies several components of thalamic neurochemical and electrophysiological remodeling after SCI: altered glutamate homeostasis, weakened presynaptic GABA_B_-receptor regulation, postsynaptic spine remodeling, increased Na_v_1.3 expression, and aberrant spontaneous and stimulus-evoked firing. These changes may collectively alter thalamic input–output conversion by acting on synaptic inputs, membrane excitability, and firing timing, thereby affecting the discrimination and transmission of sensory information below the injury level.

## 5 Thalamus-related circuits and reorganization of pain networks in the brain

The thalamus serves as both a relay station between ascending spinal sensory pathways and brain pain-processing networks and as a regulatory structure. Thalamic neurons receive spinal inputs while being regulated by inhibitory signals from corticothalamic feedback and subcortical structures such as the TRN and ZI. Bidirectional connections between different thalamic nuclei and the somatosensory cortex, insula, cingulate cortex, and prefrontal regions participate in the sensory-discriminative, attentional, affective, and behavioral dimensions of pain. Post-injury alterations in thalamus-related circuits can be considered across three interconnected domains: altered transmission of residual ascending activity from the thalamus to the cortex; changes in cortical feedback and subcortical inhibitory control over thalamic neurons; and reorganization of coupling between the thalamus and pain-modulatory structures such as the insula, cingulate cortex, and brainstem. Circuit tracing, neuronal recording, and projection-specific manipulations can validate specific pathway functions in animal models, whereas structural and functional imaging in humans is primarily used to characterize the microstructure and functional connectivity of these pathways. The principal nuclei and circuits relevant to these relationships are summarized in Figure 1.

**Figure 1:**
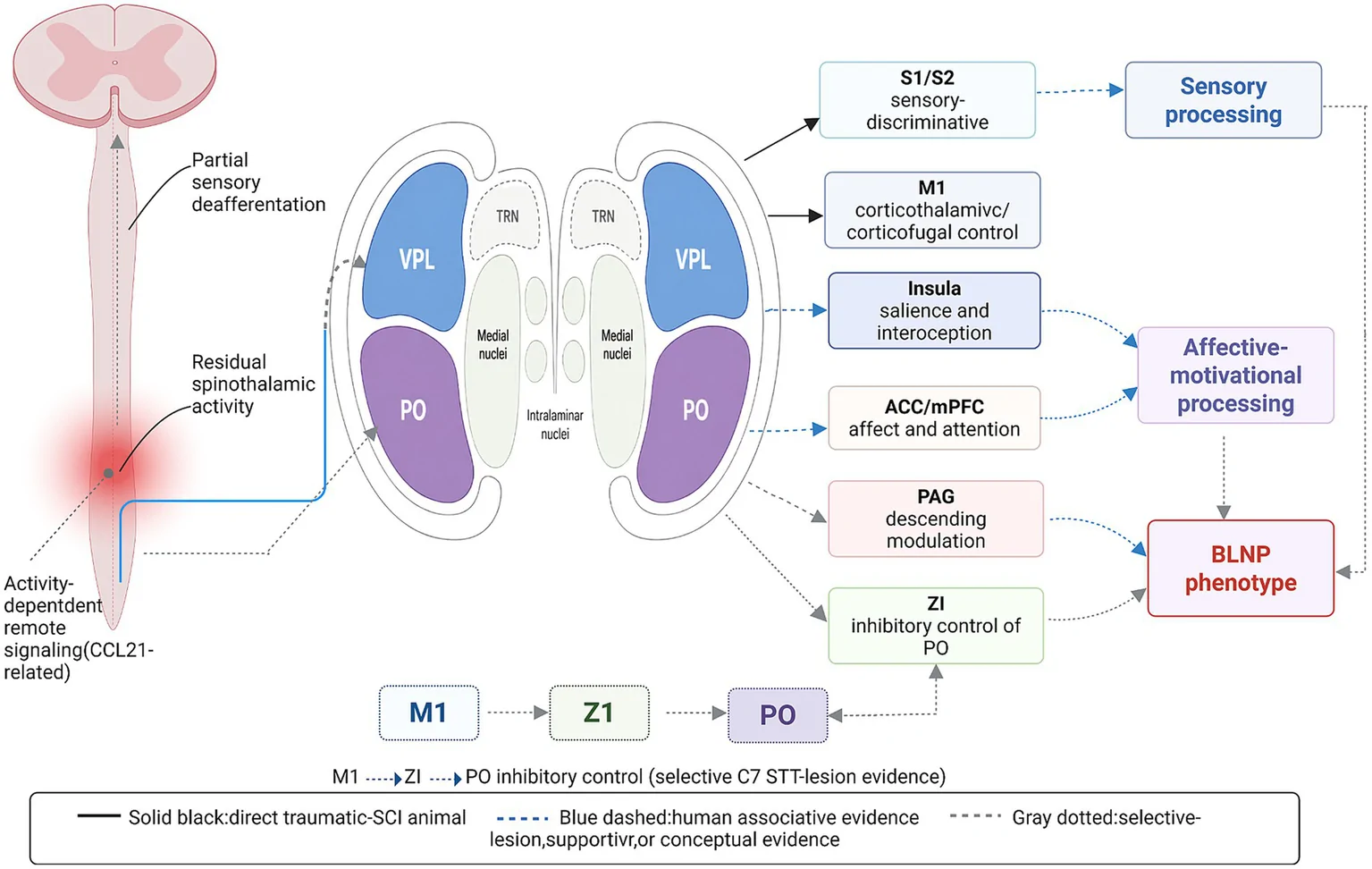
Thalamus-related nuclei and circuits relevant to pain processing after SCI. Partial sensory deafferentation, residual spinothalamic activity, and CCL21-related remote signaling are shown in relation to the VPL and PO and their connections with cortical and brainstem pain-processing regions. Solid black lines denote relationships supported by traumatic-SCI animal evidence; blue dashed lines denote human associative evidence; and gray dotted lines denote indirect, supportive, or conceptual relationships. The M1-ZI-PO inset is based on a selective C7 spinothalamic-tract lesion model and should be interpreted as indirect evidence for traumatic SCI. ACC, anterior cingulate cortex; BLNP, below-level neuropathic pain; CCL21, C-C motif chemokine ligand 21; M1, primary motor cortex; mPFC, medial prefrontal cortex; PAG, periaqueductal gray; PO, posterior thalamic nucleus; S1/S2, primary/secondary somatosensory cortex; SCI, spinal cord injury; TRN, thalamic reticular nucleus; VPL, ventral posterolateral nucleus; ZI, zona incerta.

### 5.1 Corticothalamic feedback and Thalamocortical sensory transmission

Animal studies provide projection-specific evidence that corticothalamic feedback contributes to chronic mechanical hypersensitivity following SCI. In mice entering the chronic phase after moderate spinal cord contusion, innocuous tactile stimuli abnormally enhanced neuronal activity in the lateral thalamus and multiple cortical areas. Liu et al. used a second-generation targeted active population recombination system (TRAP2) to label lateral thalamic neuron ensembles activated by tactile stimulation, followed by chemogenetic inhibition. This reduced mechanical hypersensitivity-related behavior and correspondingly decreased tactile stimulus-evoked cortical neuronal activity. Anatomical tracing revealed that after SCI, corticospinal neurons emit increased thalamus-projecting axon collaterals, accompanied by enhanced activity in related neurons. Selective ablation of corticospinal neurons could suppress the development of mechanical hypersensitivity, while inhibiting their activity even after hypersensitivity has been established still attenuated related behaviors (Liu et al., 2024). These results indicate that at both structural and functional levels, thalamic collaterals of corticospinal neurons undergo remodeling after SCI, leading to aberrant lateral thalamic responses to innocuous peripheral tactile stimuli and chronic mechanical hypersensitivity.

Human studies have examined pain-related network alterations at the level of cortical sensory representations and thalamus-related functional connectivity. Wrigley et al. compared 10 participants with complete SCI and ongoing neuropathic pain, 10 with complete SCI but no neuropathic pain, and 21 healthy controls. In the SCI group, the cortical representation evoked by little-finger touch shifted medially in the primary somatosensory cortex, and the magnitude of this shift correlated with the intensity of persistent neuropathic pain (Wrigley et al., 2009). Huynh et al. studied 19 participants with SCI and neuropathic pain, 10 with SCI but no neuropathic pain, and 25 healthy controls. Functional connectivity between the dorsomedial prefrontal cortex and the angular gyrus was stronger in the pain group than in the SCI group without neuropathic pain. Within the pain group, the bodily extent of pain showed positive correlations with connectivity between the posterior insula and multiple thalamic subregions, including the ventrolateral, mediodorsal, and posterior ventral regions (Huynh et al., 2021). A small exploratory study also reported inter-group differences in connectivity among the medial and lateral geniculate nuclei, medial and lateral pulvinar, and dorsomedial magnocellular nucleus across groups stratified by pain severity (Mandloi et al., 2024). These studies link pain intensity, body extent, and severity to cortical sensory representations and connectivity with specific thalamic subregions, but such associations do not establish specificity for pain. Karunakaran et al. also observed thalamocortical connectivity alterations in full SCI-injured patients independent of recorded pain states. Compared with 22 healthy controls, 19 subjects with complete thoracolumbar SCI exhibited weaker connectivity between the dorsomedial nucleus and the superior temporal gyrus and anterior cingulate cortex, as well as between the ventrolateral nucleus and the superior temporal gyrus. Conversely, connectivity between the pulvinar and the inferior frontal gyrus and inferior parietal lobule was stronger (Karunakaran et al., 2020). These findings establish an association between complete SCI and thalamocortical reorganization, but cannot determine whether these changes are specifically related to pain. Future research should compare subjects with and without neuropathic pain matched for neurological lesion level, injury severity, and post-injury duration, alongside detailed characterization of thalamic nuclear subdivisions as well as pain type, location, spatial extent, and intensity, to disentangle general SCI-related brain alterations from pain-specific network features.

### 5.2 Reorganization of thalamic interactions with insular, cingulate, and brainstem pain-modulatory networks

BLNP encompasses sensory-discriminative, affective, attentional, and behavioral dimensions; related functional changes may involve the insula, cingulate cortex and prefrontal cortex, and limbic-striatal networks. Li et al. (2020) compared the structural connectivity of the insular subregions in 10 participants with incomplete SCI and BLNP, 11 with incomplete SCI but without neuropathic pain, and 25 healthy controls. Relative to the non-neuropathic pain SCI patients, the BLNP group exhibited stronger connectivity between the left posterior insula and the right cerebellar lobules VIIb and VIII, as well as the occipitotemporal regions, and also showed stronger connectivity between the left ventral anterior insula and the visual association cortex. Some of these connections were positively correlated with pain scores. Compared with healthy controls, the BLNP group also demonstrated stronger connectivity between the left dorsal anterior insula and the hippocampus. The explicit definition of the below-level pain phenotype and the inclusion of pain-free SCI controls with the same injury type have provided relatively direct human evidence regarding altered functional connectivity in insular subregions. Studies using broader pain phenotypes have provided information on distributed networks. Patients with incomplete SCI and moderate-to-severe chronic pain exhibited stronger insula-frontal pole and posterior insula-anterior cingulate connectivity than healthy controls (Mandloi et al., 2023). In 37 patients with chronic SCI, neuropathic pain intensity was primarily associated with connectivity within the limbic system and between the limbic and striatal connectivity, whereas nociceptive pain intensity was more closely related to thalamocortical and thalamolimbic connectivity (Kowalski et al., 2023). Park et al. retrospectively studied 41 participants with incomplete SCI and neuropathic pain, including both at-level and below-level pain. Based on Visual Analog Scale (VAS) scores, they classified 24 individuals as having mild pain and 17 as having moderate-to-severe pain, and included 32 age- and sex-matched healthy controls. The degree and fractional amplitude of low-frequency fluctuation (fALFF) of the middle frontal gyrus node were lower in the moderate-to-severe pain group than in the mild-pain group and healthy controls. Pain intensity was positively correlated with the mean path length and negatively with fALFF in the posterior cingulate cortex (Park et al., 2023). These studies link the type, extent, and severity of pain to specific cortical and limbic-network features after SCI. Since some studies did not analyze BLNP separately, their findings mainly provide supportive evidence for changes in distributed pain networks.

Structural imaging and brainstem pain modulation studies complement the results of functional connectivity. Compared with participants with SCI but without neuropathic pain, participants with neuropathic pain showed differences in diffusion metrics within the corpus callosum, internal capsule, corona radiata, posterior thalamic radiation, cingulum, and multiple association fiber tracts (Dong et al., 2023). Another study reported higher fractional anisotropy values in the splenium of the corpus callosum and the right posterior cingulum bundle in the pain group, along with larger gray-matter volumes in the right superior and middle temporal gyri (Livny et al., 2024). Multiparametric quantitative MRI further identified differences in effective transverse relaxation rates (R_2_^*^) within the cervical spinal cord, thalamus, anterior cingulate cortex, and PAG in individuals with SCI and neuropathic pain. Pain-related thalamic R_2_^*^ differences were primarily located in the lateral thalamus, while SCI-related thalamic volume reduction involved medial regions (Kyathanahally et al., 2021). Given that these studies utilized different imaging measurement methods and pain classification criteria, their findings should not be pooled into a single interpretation suggesting either increased white-matter integrity or neurodegeneration. Within the brainstem modulatory circuits, a small study found stronger PAG connectivity with the posterior and lateral pulvinar, right mediodorsal nucleus, and ventrolateral nucleus in participants with SCI and neuropathic pain than in those with SCI but no neuropathic pain. Conversely, connectivity with the right anterior pulvinar, VPL, selected intralaminar nuclei, and geniculate bodies was weaker, indicating nucleus-dependent PAG-thalamic alterations (Shoraka et al., 2024). Conditioned pain modulation (CPM) studies have also examined descending pain control. Lütolf et al. found no significant between-group differences in supralesional CPM or temporal summation between 17 participants with SCI and neuropathic pain and 17 healthy controls, although greater spontaneous pain within the SCI group was associated with weaker inhibitory CPM (Lütolf et al., 2022). Huynh et al. similarly found that CPM efficacy was not overall reduced in 12 participants with SCI and neuropathic pain compared with 13 healthy controls, but the relationship between CPM efficacy and connectivity between the ventrolateral PAG-occipital lobe and amygdala-frontopolar differed in direction between the two groups (Huynh et al., 2023). In the latter study, higher pain intensity was associated with stronger pain inhibition, opposite to the relationship reported by Lütolf et al. Current evidence does not support attributing neuropathic pain after SCI uniformly to the loss of descending pain inhibition. Rather, CPM varies among individuals and may co-occur with altered relationships within PAG-thalamic and PAG-cortical networks.

### 5.3 Cortical-ZI regulation of thalamic activity and evidence from neuromodulation

Animal studies provide direct electrophysiological evidence that the motor cortex can regulate PO activity via the ZI. ZI is a subcortical gray-matter structure located beneath the thalamus; its ventrolateral subregion contains inhibitory neurons projecting to the PO. In rats with hypersensitivity after a unilateral C7 electrolytic spinothalamic-tract lesion, electrical stimulation of the primary motor cortex increased spontaneous activity in a subset of ventrolateral ZI neurons while simultaneously suppressing spontaneous firing in most PO neurons. Local injection of the GABA_A_-receptor agonist muscimol into the ZI reduced neuronal activity in this region, subsequently leading to the disappearance of the inhibitory response to motor-cortex stimulation in most PO neurons (Cha et al., 2013). Thus, motor-cortex stimulation inhibits PO activity at least partly via the motor cortex-ZI-PO pathway. In mice with SCI, high-frequency transcranial magnetic stimulation (TMS) applied to the primary motor cortex attenuated mechanical, cold, and thermal hypersensitivity, accompanied by enhanced motor cortex-thalamus connectivity, altered thalamic and hypothalamic transcriptomic profiles, and changes in spinal inflammatory-molecule expression (Zhao et al., 2024). These indices changed synchronously, and their relative contributions to analgesia remain to be determined. Combined with electrophysiological evidence of the motor cortex-ZI-PO pathway, these data indicate that thalamic activity after SCI is regulated not only by residual ascending inputs but also by cortical feedback and subcortical inhibitory control. Altered interactions among ascending inputs, local thalamic processing, and cortical–subcortical feedback may modulate neuronal response gain and firing timing, thereby affecting the processing of below-level sensory information within thalamocortical and brainstem pain-modulatory networks.

## 6 Discussion and future directions

### 6.1 Multilevel evidence and mechanistic positioning of thalamic remodeling

Current evidence places thalamic remodeling after SCI at three interrelated levels. First, partial sensory deafferentation can reduce normal sensory information reaching the thalamus, while incompletely interrupted ascending pathways may continue to transmit aberrant neural activity; injury-associated molecular signals such as CCL21, whose thalamic levels vary with SCI, may also alter the local cellular environment. Second, microglial responses, altered chemokine and cytokine expression, impaired excitatory-inhibitory synaptic regulation, ion-channel changes, and synaptic structural remodeling can all occur within the thalamus, modifying neuronal response gain and firing timing. Third, these local changes may be accompanied by reorganization of thalamocortical sensory transmission, corticothalamic feedback, and functional coupling between the thalamus and pain-modulatory structures such as the insula, cingulate cortex, and brainstem. These levels correspond, respectively, to the composition of thalamic inputs, local information processing, and output-feedback regulation. Therefore, thalamic remodeling after SCI should be better understood as a dynamic process spanning cellular, synaptic, and circuit scales, rather than a uniform increase in activity of a single nucleus. This multilevel evidence framework is summarized in Figure 2.

**Figure 2:**
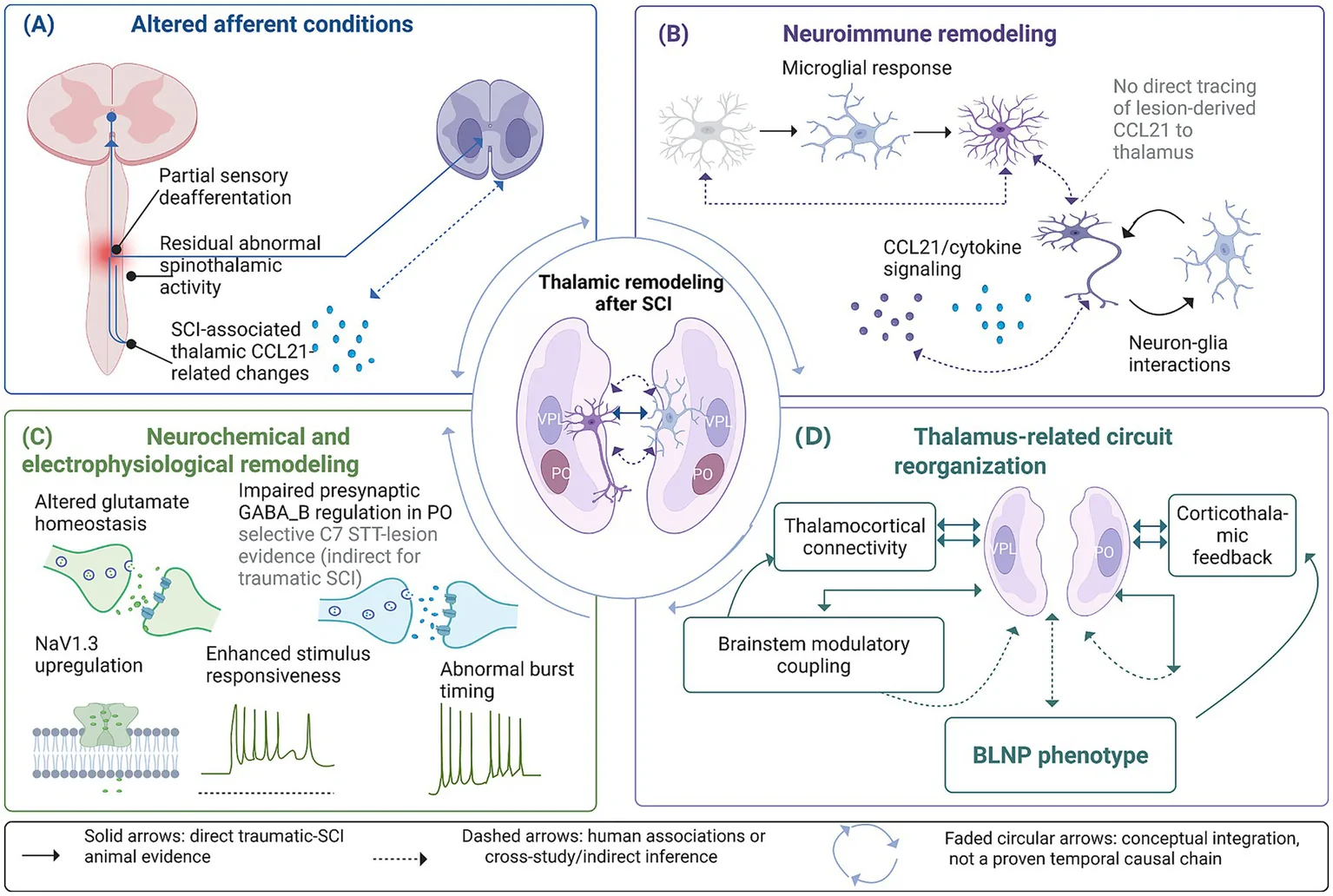
Multilevel thalamic remodeling in below-level neuropathic pain after SCI. The conceptual framework integrates **(A)** altered afferent conditions, **(B)** neuroimmune remodeling, **(C)** neurochemical and electrophysiological remodeling, and **(D)** thalamus-related circuit reorganization. Solid arrows denote direct traumatic-SCI animal evidence; dashed arrows denote human associations or cross-study/indirect inference; and faded circular arrows denote conceptual integration rather than demonstrated feedback. The diagram does not imply direct tracing of lesion-derived CCL21 to the thalamus. Evidence for altered VPL glutamate homeostasis and impaired presynaptic GABA type B receptor regulation in the PO derives from selective spinothalamic-tract lesion models and is therefore indirect for traumatic SCI. BLNP, below-level neuropathic pain; CCL21, C-C motif chemokine ligand 21; GABA, gamma-aminobutyric acid; PO, posterior thalamic nucleus; SCI, spinal cord injury; STT, spinothalamic tract; VPL, ventral posterolateral nucleus.

The relationship between thalamic changes and BLNP varies by nucleus and pain phenotype. Mechanistic evidence regarding microglial responses, impaired synaptic regulation, and stimulus-evoked enhancement is concentrated in the VPL and PO, primarily associated with behavioral manifestations of mechanical or thermal hyperalgesia. These animal behavioral metrics only reflect stimulus-evoked sensory responses and cannot fully recapitulate spontaneous pain and complex affective pain experiences in human patients. Research on medial and intralaminar nuclei has focused more on sensory integration and attention or affect networks; mechanistic evidence directly linking these nuclei to BLNP remains limited. Human studies have also shown that SCI patients without pain exhibit structural and functional thalamic alterations. Thus, nucleus-specific differences in connectivity, metabolites, and white-matter microstructure related to pain extent, intensity, or type are more informative for understanding pain-related phenotypes. Matching SCI controls for injury characteristics and applying standardized pain-phenotype stratification are critical for distinguishing general brain plasticity from pain-related thalamic changes following SCI.

Mechanistic inferences supported by different types of evidence also vary. Local molecular interventions, antisense oligonucleotides, and projection-specific manipulations in animal models can test the contribution of candidate processes to thalamic activity and pain-related behaviors. Human ^1^H-MRS, diffusion imaging, and functional-connectivity studies primarily identify biochemical, structural, or network features associated with pain phenotypes but typically cannot establish cell- or circuit-level causality in isolation. Concurrent changes in relevant metrics before and after intervention can reflect plasticity, yet their relationship to clinical efficacy still requires clarification through controlled designs, mediation analyses, and replication. This review prioritizes well-defined human studies of BLNP and animal experiments incorporating thalamic nuclei, neuronal activity, or pain-related behavior endpoints. General SCI studies without pain-subtype stratification serve mainly to provide context for describing afferent interruption, distal neuroimmune responses, and brain reorganization. Within this evidence framework, thalamic remodeling is best positioned as a potential integrative nexus among aberrant ascending inputs, altered local thalamic processing, and distributed pain-network reorganization, rather than as the sole or sufficient cause of BLNP.

### 6.2 Priorities for study design and mechanistic validation

Clinical research should establish a longitudinal observational framework extending from the subacute to chronic phases. The width of the ventral tissue bridge at 1-month post-SCI is associated with subsequent sensory function and pain outcomes (Pfyffer et al., 2020). Measures of at-level pain adaptation and CPM assessed approximately 1.5 months post-injury are also associated with the subsequent development of central neuropathic pain (Gruener et al., 2020). The Transforming Research and Clinical Knowledge in Spinal Cord Injury (TRACK-SCI) cohort recently incorporated the total number of comorbidities, Injury Severity Score, lower-extremity motor score, and total pinprick sensory score into a predictive model, achieving a balanced classification accuracy of 74.3% for chronic neuropathic pain outcomes at 6 or 12 months post-SCI (Fond et al., 2026). While this model can predict overall chronic neuropathic pain outcomes and may support clinical risk stratification, it cannot currently serve as a specific indicator of BLNP or thalamic remodeling. Future studies could repeat spinal structural imaging, quantitative sensory testing, thalamic ^1^H-MRS and perfusion imaging, nucleus-level functional-connectivity analysis, and EEG at standardized follow-up time points to elucidate the temporal relationships between thalamic metrics and pain onset, progression, and fluctuation, thereby distinguishing persistent pathological changes from phase-dependent compensatory responses.

Participants should be classified according to the International Spinal Cord Injury Pain Classification, with clear separation of BLNP, at-level neuropathic pain, nociceptive pain, and mixed pain. Studies should record neurological level and injury severity, preservation of pinprick and thermal sensation, time since injury, analgesic use, sleep, and mood. Clinical research should include at least one well-defined BLNP group, an SCI group without neuropathic pain matched for injury characteristics, and a healthy control group, while simultaneously recording pain extent, quality, triggers, and intensity. Where spatial resolution permits, thalamic imaging should utilize validated atlases or parcellation schemes to separately assess VPL, PO, intralaminar nuclei, and TRN, avoiding loss of nucleus-specific effects attributed to whole-thalamus averaging. Multicenter studies also require unified pain assessment, image acquisition, and analysis protocols, as well as prespecified primary outcome measures, to enhance reproducibility and comparability.

Basic research requires higher resolution across cell types, anatomical location, and post-injury stage. Single-cell studies of the spinal cord have demonstrated that immune-cell composition and microglial transcriptional states vary by anatomical region and time post-SCI (Fisher et al., 2022). Although this evidence derives from the injured spinal cord, the established spatiotemporal heterogeneity provides a methodological basis for investigating thalamic nuclei. Single-cell or single-nucleus transcriptomics, spatial transcriptomics, and proteomics could be applied to the VPL, PO, and intralaminar nuclei, combined with anatomical tracing of ascending spinal terminals and cortical projections. This approach could resolve cell-type- and phase-specific neuronal and glial changes within specific nuclei. Candidate circuits should be tested via cell-type- and projection-specific manipulations of the spinothalamic tract, corticothalamic tract, ZI-PO pathway, and brainstem-thalamic pathway. Behavioral assessments should extend beyond reflexive stimulus-evoked hypersensitivity to encompass spontaneous pain, avoidance, and affective behaviors, including both sexes and multiple post-injury time points, to improve correspondence with clinical pain phenotypes.

### 6.3 Candidate clinical measures and the translational potential of thalamus-related interventions

At present, thalamus-related EEG spectral and network features, thalamic metabolites and perfusion, nucleus-level functional connectivity, diffusion and structural metrics, as well as residual spinal cord tissue structure should all be considered candidate clinical markers or relevant factors. No single marker has yet been established as a biomarker for BLNP diagnosis, prognosis, or treatment-response monitoring. Their clinical value depends on prospective studies, prespecified thresholds, independent or external validation, and the ability to provide information beyond standardized pain classification. EEG offers high temporal resolution but cannot directly localize specific thalamic nuclei; ^1^H-MRS, perfusion, diffusion, and functional connectivity similarly provide regional or network-level association metrics. Patients in the subacute phase may exhibit resting-state spectral differences prior to the onset of neuropathic pain, and models based on band power have shown some capacity to classify pain onset within the next 6 months (Vuckovic et al., 2018). Nonlinear metrics such as the Higuchi fractal dimension can distinguish chronic-pain, pain-free SCI, and healthy-control groups, and show subacute features associated with subsequent pain states (Anderson et al., 2021). Connectivity at specific frequencies and lateralization differences in motor-imagery EEG also provide candidate predictive signals (Kumari et al., 2022, 2023), but all markers require external validation before clinical application.

Chronic-phase studies further distinguish between EEG features related to injury and those related to pain. In SCI participants with pain, theta-band connectivity changes and local efficiency in the frontoparietal network were more pronounced, while beta- and gamma-band changes in the sensorimotor network were evident in both painful and pain-free SCI groups, potentially reflecting SCI itself more broadly (Hasan et al., 2024). Compared with individuals with SCI numbness but no neuropathic pain, the pain group exhibited lower theta- and alpha-band power, higher beta-band power, and corresponding connectivity differences; high-gamma oscillations and network features in the prefrontal region were also associated with pain symptoms (Wang et al., 2024, 2025). However, testing on independent datasets reduced classifier accuracy from over 80% in single datasets to 66.6%, highlighting limitations in cross-sample generalizability (Anderson et al., 2025). A small longitudinal home-based study found that EEG power and reactivity indices varied with pain intensity, providing preliminary support for state monitoring (Nawaz et al., 2024). Integrating EEG with thalamic ^1^H-MRS, perfusion, nucleus-level connectivity, and spinal tissue-bridge metrics may support multimodal models with mechanistic interpretability, though independent and multicenter validation remains necessary.

Noninvasive brain stimulation may modulate post-SCI pain through cortical excitability and thalamus-related networks, although current efficacy assessments yield inconsistent results. In a randomized study involving 37 SCI participants, intermittent theta-burst stimulation, 10-Hz repetitive TMS, or sequential application of both resulted in decreased pain scores, with varying degrees of improvement across active protocols (Yang et al., 2023). As the trial compared active stimulation regimens rather than adequate sham conditions, it primarily serves as a reference for parameter selection and cannot replace sham-controlled efficacy trials. TMS assessment also revealed lower motor-evoked potentials in the hand areas of both primary motor cortices in people with SCI and neuropathic pain; interhemispheric excitatory asymmetry was associated with pain duration, intensity, and emotional distress (Liu et al., 2025). Meta-analyses on the overall analgesic effects of non-invasive brain stimulation have reached divergent conclusions, likely attributed to variations in pain classification, target sites and stimulation parameters, control conditions, and follow-up durations (Yu et al., 2020; Li et al., 2022). Future trials should clearly define BLNP, include adequate sham conditions, and assess prespecified mechanistic outcomes, such as thalamic GABA-related signaling, regional perfusion, nucleus-level connectivity, and EEG rhythms, in addition to pain endpoints, to determine whether interventions modulate prespecified thalamocortical mechanisms.

Sensorimotor and multisensory rehabilitation provides another line of evidence regarding the modifiability of brain networks. In a small single-group pre-post intervention study, participants with complete thoracic SCI and persistent BLNP showed reduced pain scores and increased thalamic GABA/Cr following immersive virtual walking, though the magnitude of these two changes was not significantly correlated (Gustin et al., 2023). In a randomized controlled trial, immersive virtual motor training reduced neuropathic pain scores more effectively than a two-dimensional video condition and improved symptoms related to depersonalization (Selvaraj et al., 2026). Cognitive multisensory rehabilitation also alleviated SCI-associated neuropathic pain and improved sensorimotor function, accompanied by altered resting-state connectivity in the parietal operculum and task-related brain activity changes (Van de Winckel et al., 2026). The latter two studies did not directly measure the thalamus; they support clinical effects and brain-network plasticity but do not establish the thalamus’s contribution to treatment response. Future research could pre-specify thalamic metabolites, perfusion, and thalamocortical connectivity as mechanistic outcomes and examine their temporal and dose–response relationships with clinical improvement.

Invasive interventions provide exploratory evidence regarding the reversibility of supraspinal pain networks and the candidate target selection. Eight patients with clinically complete SCI and refractory BLNP achieved pain relief following microcoagulation of the dorsal root entry zone, accompanied by altered connectivity in sensorimotor, medial prefrontal, and limbic networks (Falci et al., 2024). A subsequent study of 24 patients found that preoperative white-matter microstructure, gray-matter morphology, and PAG-sensorimotor connectivity varied according to surgical outcomes (Chen et al., 2026). Since the targets were located in the spinal cord, these studies suggest that reducing aberrant spinal input may be accompanied by supraspinal network changes; however, this is not direct evidence of thalamic intervention. A diffusion-imaging study proposed a computational method for identifying candidate deep-brain-stimulation targets from the splenium of the corpus callosum and its connected fibers, though the clinical efficacy of these targets remains unverified (Black et al., 2022). A phase I clinical trial of magnetic resonance-guided focused ultrasound central lateral thalamotomy reported improvements in pain and function and altered default-mode-network connectivity in patients with refractory neuropathic pain of mixed etiologies (Ahmed et al., 2024). This small, uncontrolled, heterogeneous-etiology study provides preliminary information on the feasibility and safety of direct thalamic intervention, but is insufficient to establish efficacy for post-SCI BLNP. The thalamus and its connected networks may hold value for target selection and treatment stratification, but translational success requires precise pain phenotyping, nucleus-specific functional localization, controlled trials, and long-term safety assessments.

### 6.4 Conclusion

Following SCI, local inflammation within the injured spinal cord, neuronal hyperexcitability, and central sensitization constitute important pathological bases for BLNP, yet they are insufficient to explain its long-term maintenance and clinical heterogeneity. Existing evidence supports a multilevel view of thalamic remodeling after SCI: partial sensory deafferentation, abnormal neural activity transmitted via spared ascending pathways, and molecular signals related to spinal conduction alter thalamic afferent conditions and local cellular environments. Microglial responses, abnormal excitatory-inhibitory synaptic regulation, changes in ion-channel expression, and synaptic structural alterations further influence the response magnitude, stimulus selectivity, and firing timing of thalamic neurons. Corticothalamic feedback and functional reorganization between the thalamus and somatosensory cortex, insula, cingulate cortex, and brainstem pain-modulatory structures link local thalamic changes to widespread brain pain networks. These alterations vary by nucleus, cell type, and stage of injury. More direct animal mechanism evidence focuses primarily on the VPL and PO, while current human studies mainly reveal associations between thalamic metabolites, perfusion, microstructure, and functional connectivity with pain phenotypes. Future research should employ standardized pain classification, injury-matched pain-free SCI controls, thalamic nuclear parcellation, and longitudinal multimodal designs, combined with cell- and projection-specific validation in animal experiments. Only imaging or EEG markers that have been evaluated against prespecified thresholds and replicated in independent cohorts can transition from candidate clinical markers to usable biomarkers.

## References

[ref1] Ahmed A. K. Zhuo J. Gullapalli R. P. Jiang L. Keaser M. L. Greenspan J. D. . (2024). Focused ultrasound central lateral thalamotomy for the treatment of refractory neuropathic pain: phase I trial. Neurosurgery 94, 690–699. doi: 10.1227/neu.0000000000002752, 37947407

[ref2] Anderson K. Chirion C. Fraser M. Purcell M. Stein S. Vuckovic A. (2021). Markers of central neuropathic pain in Higuchi fractal analysis of EEG signals from people with spinal cord injury. Front. Neurosci. 15:705652. doi: 10.3389/fnins.2021.705652, 34512243 PMC8427815

[ref3] Anderson K. Stein S. Suen H. Purcell M. Belci M. McCaughey E. . (2025). Generalisation of EEG-based pain biomarker classification for predicting central neuropathic pain in subacute spinal cord injury. Biomedicine 13:213. doi: 10.3390/biomedicines13010213, 39857795 PMC11759196

[ref4] Awad A. Levi R. Waller M. Westling G. Lindgren L. Eriksson J. (2020). Preserved somatosensory conduction in complete spinal cord injury: discomplete SCI. Clin. Neurophysiol. 131, 1059–1067. doi: 10.1016/j.clinph.2020.01.017, 32197128

[ref5] Biber K. Tsuda M. Tozaki-Saitoh H. Tsukamoto K. Toyomitsu E. Masuda T. . (2011). Neuronal CCL21 up-regulates microglia P2X4 expression and initiates neuropathic pain development. EMBO J. 30, 1864–1873. doi: 10.1038/emboj.2011.89, 21441897 PMC3101996

[ref6] Black S. R. Janson A. Mahan M. Anderson J. Butson C. R. (2022). Identification of deep brain stimulation targets for neuropathic pain after spinal cord injury using localized increases in white matter fiber cross section. Neuromodulation 25, 276–285. doi: 10.1111/ner.13399, 35125147

[ref7] Brown E. V. Malik A. F. Moese E. R. McElroy A. F. Lepore A. C. (2022). Differential activation of pain circuitry neuron populations in a mouse model of spinal cord injury-induced neuropathic pain. J. Neurosci. 42, 3271–3289. doi: 10.1523/JNEUROSCI.1596-21.2022, 35256528 PMC8994550

[ref8] Bryce T. N. Biering-Sørensen F. Finnerup N. B. Cardenas D. D. Defrin R. Lundeberg T. . (2012). International spinal cord injury pain classification: part I. Background and description. Spinal Cord 50, 413–417. doi: 10.1038/sc.2011.156, 22182852

[ref9] Bryce T. N. Tsai C. Y. Dyson-Hudson T. Felix E. R. Haubert L. L. Weir J. . (2026). Patterns of pain and nonpainful sensations experienced during the first 12 months after spinal cord injury. Arch. Phys. Med. Rehabil. 107, 1657–1669. doi: 10.1016/j.apmr.2025.11.024, 41360352

[ref10] Calderone A. Cardile D. De Luca R. Quartarone A. Corallo F. Calabrò R. S. (2024). Brain plasticity in patients with spinal cord injuries: a systematic review. Int. J. Mol. Sci. 25:2224. doi: 10.3390/ijms25042224, 38396902 PMC10888628

[ref11] Castany S. Bagó-Mas A. Vela J. M. Verdú E. Bretová K. Svobodová V. . (2023). Transient reflexive pain responses and chronic affective nonreflexive pain responses associated with neuroinflammation processes in both spinal and supraspinal structures in spinal cord-injured female mice. Int. J. Mol. Sci. 24:1761. doi: 10.3390/ijms24021761, 36675275 PMC9863935

[ref12] Cha M. Ji Y. Masri R. (2013). Motor cortex stimulation activates the incertothalamic pathway in an animal model of spinal cord injury. J. Pain 14, 260–269. doi: 10.1016/j.jpain.2012.11.007, 23332495 PMC3594418

[ref13] Chen Q. Li X. Wang L. Chen Z. Zhang Y. Becker L. . (2026). Distinct preoperative multimodal brain reorganizations underlie differential outcomes of DREZ lesioning for neuropathic pain after spinal cord injury. Brain Res. Bull. 241:111970. doi: 10.1016/j.brainresbull.2026.111970, 42202434

[ref14] Colloca L. Ludman T. Bouhassira D. Baron R. Dickenson A. H. Yarnitsky D. . (2017). Neuropathic pain. Nat. Rev. Dis. Primers 3:17002. doi: 10.1038/nrdp.2017.2, 28205574 PMC5371025

[ref15] de Jong E. K. Dijkstra I. M. Hensens M. Brouwer N. van Amerongen M. Liem R. S. . (2005). Vesicle-mediated transport and release of CCL21 in endangered neurons: a possible explanation for microglia activation remote from a primary lesion. J. Neurosci. 25, 7548–7557. doi: 10.1523/JNEUROSCI.1019-05.2005 PMC6725403 16107642

[ref16] de Jong E. K. Vinet J. Stanulovic V. S. Meijer M. Wesseling E. Sjollema K. . (2008). Expression, transport, and axonal sorting of neuronal CCL21 in large dense-core vesicles. FASEB J. 22, 4136–4145. doi: 10.1096/fj.07-101907, 18697841

[ref17] Detloff M. R. Fisher L. C. McGaughy V. Longbrake E. E. Popovich P. G. Basso D. M. (2008). Remote activation of microglia and pro-inflammatory cytokines predict the onset and severity of below-level neuropathic pain after spinal cord injury in rats. Exp. Neurol. 212, 337–347. doi: 10.1016/j.expneurol.2008.04.009, 18511041 PMC2600773

[ref18] Dong D. Hosomi K. Mori N. Kamijo Y. I. Furotani Y. Yamagami D. . (2023). White matter microstructural alterations in patients with neuropathic pain after spinal cord injury: a diffusion tensor imaging study. Front. Neurol. 14:1241658. doi: 10.3389/fneur.2023.1241658, 37693753 PMC10484711

[ref19] Falci S. Morse L. Berliner J. Murakami M. Welch A. Barnkow D. . (2024). Neuropathic pain relief and altered brain networks after dorsal root entry zone microcoagulation in patients with spinal cord injury. Brain Commun. 6:fcae411. doi: 10.1093/braincomms/fcae411, 39605971 PMC11601164

[ref20] Ferreira T. Cruz C. D. Costa-Pereira J. T. (2026). Role of supraspinal neuroinflammation in chronic pain after experimental spinal cord injury-a systematic review. Int. J. Mol. Sci. 27:5711. doi: 10.3390/ijms27135711, 42449982 PMC13361473

[ref21] Fisher E. S. Amarante M. A. Lowry N. Lotz S. Farjood F. Temple S. . (2022). Single cell profiling of CD45+ spinal cord cells reveals microglial and B cell heterogeneity and crosstalk following spinal cord injury. J. Neuroinflammation 19:266. doi: 10.1186/s12974-022-02627-3, 36333772 PMC9635187

[ref22] Fond K. A. Arellano M. Torres-Espin A. Chou A. Bradfield X. Moncivais S. L. . (2026). Acute injury characteristics predict chronic neuropathic pain development after spinal cord injury. Front. Neurol. 17:1814624. doi: 10.3389/fneur.2026.1814624, 42383031 PMC13314459

[ref23] Fukushi R. Sasaki M. Obara H. Kurihara K. Hirota R. Morita T. . (2026). Mesenchymal stem cells reverse disease-specific abnormalities in nociceptive regions of the brain. Brain Commun. 8:fcaf494. doi: 10.1093/braincomms/fcaf494, 41509715 PMC12776362

[ref24] Gerke M. B. Duggan A. W. Xu L. Siddall P. J. (2003). Thalamic neuronal activity in rats with mechanical allodynia following contusive spinal cord injury. Neuroscience 117, 715–722. doi: 10.1016/s0306-4522(02)00961-2, 12617975

[ref25] Gruener H. Zeilig G. Gaidukov E. Rachamim-Katz O. Ringler E. Blumen N. . (2020). Biomarkers for predicting central neuropathic pain occurrence and severity after spinal cord injury: results of a long-term longitudinal study. Pain 161, 545–556. doi: 10.1097/j.pain.0000000000001740, 31693542

[ref26] Gustin S. M. Bolding M. Willoughby W. Anam M. Shum C. Rumble D. . (2023). Cortical mechanisms underlying immersive interactive virtual walking treatment for amelioration of neuropathic pain after spinal cord injury: findings from a preliminary investigation of thalamic inhibitory function. J. Clin. Med. 12:5743. doi: 10.3390/jcm12175743, 37685810 PMC10488675

[ref27] Gustin S. M. Wrigley P. J. Youssef A. M. McIndoe L. Wilcox S. L. Rae C. D. . (2014). Thalamic activity and biochemical changes in individuals with neuropathic pain after spinal cord injury. Pain 155, 1027–1036. doi: 10.1016/j.pain.2014.02.008, 24530612 PMC4410007

[ref28] Gwak Y. S. Kim H. K. Kim H. Y. Leem J. W. (2010). Bilateral hyperexcitability of thalamic VPL neurons following unilateral spinal injury in rats. J. Physiol. Sci. 60, 59–66. doi: 10.1007/s12576-009-0066-2, 19882377 PMC10717679

[ref29] Hains B. C. Saab C. Y. Waxman S. G. (2005). Changes in electrophysiological properties and sodium channel Nav1.3 expression in thalamic neurons after spinal cord injury. Brain 128, 2359–2371. doi: 10.1093/brain/awh623, 16109750

[ref30] Hains B. C. Saab C. Y. Waxman S. G. (2006). Alterations in burst firing of thalamic VPL neurons and reversal by Na(v)1.3 antisense after spinal cord injury. J. Neurophysiol. 95, 3343–3352. doi: 10.1152/jn.01009.2005, 16481457

[ref31] Hasan M. A. Sattar P. Qazi S. A. Fraser M. Vuckovic A. (2024). Brain networks with modified connectivity in patients with neuropathic pain and spinal cord injury. Clin. EEG Neurosci. 55, 88–100. doi: 10.1177/15500594211051485, 34714181

[ref32] He Z. Zhang J. Xu J. Wang Y. Zheng X. Wang W. (2025). Differential neuronal activation of nociceptive pathways in neuropathic pain after spinal cord injury. Cell. Mol. Neurobiol. 45:18. doi: 10.1007/s10571-025-01532-6, 39883258 PMC11782389

[ref33] Ho T. Holle J. Limakatso K. Ryan M. Tang S. J. Gustin S. M. . (2026). Functional brain network alterations in neuropathic pain after spinal cord injury: a systematic review. Neurosci. Biobehav. Rev. 188:106794. doi: 10.1016/j.neubiorev.2026.106794, 42242479

[ref34] Hubscher C. H. Johnson R. D. (2006). Chronic spinal cord injury induced changes in the responses of thalamic neurons. Exp. Neurol. 197, 177–188. doi: 10.1016/j.expneurol.2005.09.007, 16266704

[ref35] Hunt C. Moman R. Peterson A. Wilson R. Covington S. Mustafa R. . (2021). Prevalence of chronic pain after spinal cord injury: a systematic review and meta-analysis. Reg. Anesth. Pain Med. 46, 328–336. doi: 10.1136/rapm-2020-101960, 33408161

[ref36] Huynh V. Lütolf R. Rosner J. Luechinger R. Curt A. Kollias S. . (2021). Supraspinal nociceptive networks in neuropathic pain after spinal cord injury. Hum. Brain Mapp. 42, 3733–3749. doi: 10.1002/hbm.25401, 34132441 PMC8288099

[ref37] Huynh V. Lütolf R. Rosner J. Luechinger R. Curt A. Kollias S. . (2023). Intrinsic brain connectivity alterations despite intact pain inhibition in subjects with neuropathic pain after spinal cord injury: a pilot study. Sci. Rep. 13:11943. doi: 10.1038/s41598-023-37783-w, 37488130 PMC10366123

[ref38] Huynh V. Rosner J. Curt A. Kollias S. Hubli M. Michels L. (2020). Disentangling the effects of spinal cord injury and related neuropathic pain on supraspinal neuroplasticity: a systematic review on neuroimaging. Front. Neurol. 10:1413. doi: 10.3389/fneur.2019.01413, 32116986 PMC7013003

[ref39] Kang J. Cho S. S. Kim H. Y. Lee B. H. Cho H. J. Gwak Y. S. (2020). Regional hyperexcitability and chronic neuropathic pain following spinal cord injury. Cell. Mol. Neurobiol. 40, 861–878. doi: 10.1007/s10571-020-00785-7, 31955281 PMC11448802

[ref40] Karunakaran K. D. Yuan R. He J. Zhao J. Cui J. L. Zang Y. F. . (2020). Resting-state functional connectivity of the thalamus in complete spinal cord injury. Neurorehabil. Neural Repair 34, 122–133. doi: 10.1177/1545968319893299, 31904298

[ref41] Kowalski J. L. Morse L. R. Troy K. Nguyen N. Battaglino R. A. Falci S. P. . (2023). Resting state functional connectivity differentiation of neuropathic and nociceptive pain in individuals with chronic spinal cord injury. Neuroimage Clin. 38:103414. doi: 10.1016/j.nicl.2023.103414, 37244076 PMC10238876

[ref42] Kubota A. Nakajima H. Honjoh K. Watanabe S. Takahashi A. Matsumine A. (2026). Differential microglial dynamics and neuroinflammation underlying neuropathic pain in the central nervous system: comparative insights from spinal cord injury and compressive myelopathy models. Front. Cell. Neurosci. 20:1769004. doi: 10.3389/fncel.2026.1769004, 41660107 PMC12875993

[ref43] Kumari R. Gibson H. Jarjees M. Turner C. Purcell M. Vučković A. (2023). The predictive value of cortical activity during motor imagery for subacute spinal cord injury-induced neuropathic pain. Clin. Neurophysiol. 148, 32–43. doi: 10.1016/j.clinph.2023.01.006, 36796284

[ref44] Kumari R. Jarjees M. Susnoschi-Luca I. Purcell M. Vučković A. (2022). Effective connectivity in spinal cord injury-induced neuropathic pain. Sensors 22:6337. doi: 10.3390/s22176337, 36080805 PMC9460641

[ref45] Kyathanahally S. P. Azzarito M. Rosner J. Calhoun V. D. Blaiotta C. Ashburner J. . (2021). Microstructural plasticity in nociceptive pathways after spinal cord injury. J. Neurol. Neurosurg. Psychiatry 92, 863–871. doi: 10.1136/jnnp-2020-325580, 34039630 PMC8292587

[ref46] Lee J. Y. Park C. S. Seo K. J. Kim I. Y. Han S. Youn I. . (2023). IL-6/JAK2/STAT3 axis mediates neuropathic pain by regulating astrocyte and microglia activation after spinal cord injury. Exp. Neurol. 370:114576. doi: 10.1016/j.expneurol.2023.114576, 37863306

[ref47] Li L. Huang H. Yu Y. Jia Y. Liu Z. Shi X. . (2022). Non-invasive brain stimulation for neuropathic pain after spinal cord injury: a systematic review and network meta-analysis. Front. Neurosci. 15:800560. doi: 10.3389/fnins.2021.800560, 35221889 PMC8873374

[ref48] Li X. Wang L. Chen Q. Hu Y. Du J. Chen X. . (2020). The reorganization of insular subregions in individuals with below-level neuropathic pain following incomplete spinal cord injury. Neural Plast. 2020:2796571. doi: 10.1155/2020/2796571, 32211038 PMC7085828

[ref49] Liang L. Mendell L. M. (2013). Bilateral transient changes in thalamic nucleus ventroposterior lateralis after thoracic hemisection in the rat. J. Neurophysiol. 110, 942–951. doi: 10.1152/jn.00998.2012, 23741041 PMC3742973

[ref50] Liu X. Dai C. Gao M. Lin X. Xi X. Wu X. . (2025). Corticospinal excitability in bilateral M1 hand areas: association with neuropathic pain after spinal cord injury. J. Pain Res. 18, 4003–4018. doi: 10.2147/JPR.S517353, 40822430 PMC12356214

[ref51] Liu L. Liang Z. Zhang L. Feng Z. Cao F. Zhang Y. . (2024). Corticothalamic input derived from corticospinal neurons contributes to chronic neuropathic pain after spinal cord injury. Exp. Neurol. 381:114923. doi: 10.1016/j.expneurol.2024.114923, 39142366

[ref52] Livny A. Golan Y. Itzhaki N. Grossberg D. Tsarfaty G. Bondi M. . (2024). Higher regional gray matter volume and white matter integrity in individuals with central neuropathic pain after spinal cord injury. J. Neurotrauma 41, 836–843. doi: 10.1089/neu.2023.0146, 37937697

[ref53] Lütolf R. De Schoenmacker I. Rosner J. Sirucek L. Schweinhardt P. Curt A. . (2022). Anti- and pro-nociceptive mechanisms in neuropathic pain after human spinal cord injury. Eur. J. Pain 26, 2176–2187. doi: 10.1002/ejp.2029, 36000307 PMC9826499

[ref54] Mandloi S. Syed M. Ailes I. Shoraka O. Leiby B. Miao J. . (2024). Exploring functional connectivity in chronic spinal cord injury patients with neuropathic pain versus without neuropathic pain. Neurotrauma Rep. 5, 16–27. doi: 10.1089/neur.2023.0070, 38249324 PMC10797176

[ref55] Mandloi S. Syed M. Shoraka O. Ailes I. Kang K. C. Sathe A. . (2023). The role of the insula in chronic pain following spinal cord injury: a resting-state fMRI study. J. Neuroimaging 33, 781–791. doi: 10.1111/jon.13117, 37188633

[ref56] Mandwie M. Piper J. A. Gorrie C. A. Keay K. A. Musumeci G. Al-Badri G. . (2022). Rapid GFAP and Iba1 expression changes in the female rat brain following spinal cord injury. Neural Regen. Res. 17, 378–385. doi: 10.4103/1673-5374.317982, 34269213 PMC8463994

[ref57] Naderi A. Asgari A. R. Zahed R. Ghanbari A. Samandari R. Jorjani M. (2014). Estradiol attenuates spinal cord injury-related central pain by decreasing glutamate levels in thalamic VPL nucleus in male rats. Metab. Brain Dis. 29, 763–770. doi: 10.1007/s11011-014-9570-z, 24879046

[ref58] Nawaz R. Suen H. Ullah R. Purcell M. Diggin S. McCaughey E. . (2024). Electroencephalography longitudinal markers of central neuropathic pain intensity in spinal cord injury: a home-based pilot study. Biomedicine 12:2751. doi: 10.3390/biomedicines12122751, 39767658 PMC11672874

[ref59] Park A. Li Y. Masri R. Keller A. (2017). Presynaptic and extrasynaptic regulation of posterior nucleus of thalamus. J. Neurophysiol. 118, 507–519. doi: 10.1152/jn.00862.2016, 28331010 PMC5511867

[ref60] Park E. Park J. W. Kim E. Min Y. S. Lee H. J. Jung T. D. . (2023). Effects of alterations in resting-state neural networks on the severity of neuropathic pain after spinal cord injury. Bioengineering 10:860. doi: 10.3390/bioengineering10070860, 37508887 PMC10376439

[ref61] Park A. Uddin O. Li Y. Masri R. Keller A. (2018). Pain after spinal cord injury is associated with abnormal presynaptic inhibition in the posterior nucleus of the thalamus. J. Pain 19, 727.e1–727.e15. doi: 10.1016/j.jpain.2018.02.002, 29481977 PMC6026070

[ref62] Pattany P. M. Yezierski R. P. Widerström-Noga E. G. Bowen B. C. Martinez-Arizala A. Garcia B. R. . (2002). Proton magnetic resonance spectroscopy of the thalamus in patients with chronic neuropathic pain after spinal cord injury. AJNR Am. J. Neuroradiol. 23, 901–905. PMC7976918 12063213

[ref63] Pfyffer D. Vallotton K. Curt A. Freund P. (2020). Tissue bridges predict neuropathic pain emergence after spinal cord injury. J. Neurol. Neurosurg. Psychiatry 91, 1111–1117. doi: 10.1136/jnnp-2020-323150, 32788257 PMC7509517

[ref64] Ramos D. Cruz C. D. (2023). Involvement of microglia in chronic neuropathic pain associated with spinal cord injury - a systematic review. Rev. Neurosci. 34, 933–950. doi: 10.1515/revneuro-2023-0031, 37490300

[ref65] Rosner J. de Andrade D. C. Davis K. D. Gustin S. M. Kramer J. L. K. Seal R. P. . (2023). Central neuropathic pain. Nat. Rev. Dis. Primers 9:73. doi: 10.1038/s41572-023-00484-9, 38129427 PMC11329872

[ref66] Saghaei E. Abbaszadeh F. Naseri K. Ghorbanpoor S. Afhami M. Haeri A. . (2013). Estradiol attenuates spinal cord injury-induced pain by suppressing microglial activation in thalamic VPL nuclei of rats. Neurosci. Res. 75, 316–323. doi: 10.1016/j.neures.2013.01.010, 23419864

[ref67] Salehian F. Aarabi A. Rajai Firouzabadi S. Alinejadfard M. Oraee S. Mohammadi I. . (2025). Prevalence of neuropathic pain following spinal cord injury: an updated systematic review and meta-analysis. J. Clin. Neurosci. 142:111660. doi: 10.1016/j.jocn.2025.111660, 41043376

[ref68] Selvaraj J. S. Prakash H. Augustine T. A. Kumar S. S. K. Velkumar S. Amalraj J. A. (2026). Immersive virtual reality for neuropathic pain management in spinal cord injury: a randomized controlled trial. J. Spinal Cord Med. 49, 543–555. doi: 10.1080/10790268.2025.2514321, 40638262 PMC13123074

[ref69] Shoraka O. Syed M. Mandloi S. Thalheimer S. Kashani S. N. Heller J. E. . (2024). Periaqueductal gray connectivity in spinal cord injury-induced neuropathic pain. J. Neuroimaging 34, 704–719. doi: 10.1111/jon.13237, 39252511 PMC12619528

[ref70] Siddall P. J. McClelland J. M. Rutkowski S. B. Cousins M. J. (2003). A longitudinal study of the prevalence and characteristics of pain in the first 5 years following spinal cord injury. Pain 103, 249–257. doi: 10.1016/S0304-3959(02)00452-9, 12791431

[ref71] Siddall P. J. Taylor D. A. McClelland J. M. Rutkowski S. B. Cousins M. J. (1999). Pain report and the relationship of pain to physical factors in the first 6 months following spinal cord injury. Pain 81, 187–197. doi: 10.1016/s0304-3959(99)00023-8, 10353507

[ref72] Tan M. Feng Z. Chen H. Min L. Wen H. Liu H. . (2023). Transcranial direct current stimulation regulates phenotypic transformation of microglia to relieve neuropathic pain induced by spinal cord injury. Front. Behav. Neurosci. 17:1147693. doi: 10.3389/fnbeh.2023.1147693, 37081929 PMC10110883

[ref73] Van de Winckel A. Carpentier S. T. Deng W. Bottale S. Zhang L. Hendrickson T. J. . (2026). Preliminary efficacy of cognitive multisensory rehabilitation for neuropathic pain in chronic spinal cord injury: a phase I randomized controlled trial. Sci. Rep. 16:14330. doi: 10.1038/s41598-026-44859-w, 41862607 PMC13144352

[ref74] Vuckovic A. Gallardo V. J. F. Jarjees M. Fraser M. Purcell M. (2018). Prediction of central neuropathic pain in spinal cord injury based on EEG classifier. Clin. Neurophysiol. 129, 1605–1617. doi: 10.1016/j.clinph.2018.04.750, 29886266

[ref75] Wang G. Thompson S. M. (2008). Maladaptive homeostatic plasticity in a rodent model of central pain syndrome: thalamic hyperexcitability after spinothalamic tract lesions. J. Neurosci. 28, 11959–11969. doi: 10.1523/JNEUROSCI.3296-08.2008, 19005061 PMC2627563

[ref76] Wang D. Zhang X. Xin C. Wang C. Yue S. Guo D. . (2024). Electroencephalography-based biological and functional characteristics of spinal cord injury patients with neuropathic pain and numbness. Front. Neurosci. 18:1356858. doi: 10.3389/fnins.2024.1356858, 38751860 PMC11094546

[ref77] Wang D. Zhang X. Yue S. Guo D. Jiang L. Feng C. . (2025). Analysis of electroencephalography oscillation characteristics in spinal cord injury patients with neuropathic pain. Brain Res. Bull. 229:111438. doi: 10.1016/j.brainresbull.2025.111438, 40581333

[ref78] Widerström-Noga E. (2023). Neuropathic pain and spinal cord injury: management, phenotypes, and biomarkers. Drugs 83, 1001–1025. doi: 10.1007/s40265-023-01903-7, 37326804

[ref79] Widerström-Noga E. Cruz-Almeida Y. Felix E. R. Pattany P. M. (2015). Somatosensory phenotype is associated with thalamic metabolites and pain intensity after spinal cord injury. Pain 156, 166–174. doi: 10.1016/j.pain.0000000000000019, 25599312 PMC4423177

[ref80] Wrigley P. J. Press S. R. Gustin S. M. Macefield V. G. Gandevia S. C. Cousins M. J. . (2009). Neuropathic pain and primary somatosensory cortex reorganization following spinal cord injury. Pain 141, 52–59. doi: 10.1016/j.pain.2008.10.007, 19027233

[ref81] Wu J. Raver C. Piao C. Keller A. Faden A. I. (2013). Cell cycle activation contributes to increased neuronal activity in the posterior thalamic nucleus and associated chronic hyperesthesia after rat spinal cord contusion. Neurotherapeutics 10, 520–538. doi: 10.1007/s13311-013-0198-1, 23775067 PMC3701760

[ref82] Wydenkeller S. Maurizio S. Dietz V. Halder P. (2009). Neuropathic pain in spinal cord injury: significance of clinical and electrophysiological measures. Eur. J. Neurosci. 30, 91–99. doi: 10.1111/j.1460-9568.2009.06801.x, 19558605

[ref83] Yang C. Bi Y. Hu L. Gong L. Li Z. Zhang N. . (2023). Effects of different transcranial magnetic stimulations on neuropathic pain after spinal cord injury. Front. Neurol. 14:1141973. doi: 10.3389/fneur.2023.1141973, 37521294 PMC10374342

[ref84] Yu H. Chen D. Jiang H. Fu G. Yang Y. Deng Z. . (2022). Brain morphology changes after spinal cord injury: a voxel-based meta-analysis. Front. Neurol. 13:999375. doi: 10.3389/fneur.2022.999375, 36119697 PMC9477418

[ref85] Yu B. Qiu H. Li J. Zhong C. Li J. (2020). Noninvasive brain stimulation does not improve neuropathic pain in individuals with spinal cord injury: evidence from a meta-analysis of 11 randomized controlled trials. Am. J. Phys. Med. Rehabil. 99, 811–820. doi: 10.1097/PHM.0000000000001421, 32175926

[ref86] Zhao P. Waxman S. G. Hains B. C. (2007). Modulation of thalamic nociceptive processing after spinal cord injury through remote activation of thalamic microglia by cysteine cysteine chemokine ligand 21. J. Neurosci. 27, 8893–8902. doi: 10.1523/JNEUROSCI.2209-07.2007, 17699671 PMC6672166

[ref87] Zhao Q. Zhao L. Fan P. Zhu Y. Zhu R. Cheng L. . (2024). Non-invasive TMS attenuates neuropathic pain after spinal cord injury associated with enhancing brain functional connectivity and HPA axis activity. Heliyon 10:e36061. doi: 10.1016/j.heliyon.2024.e36061, 39253232 PMC11382048

