# Supplementary material for: Thalamic remodeling underlying below-level neuropathic pain following spinal cord injury: neuroimmune, neurochemical, and circuit-level mechanisms
Source: Front Cell Neurosci. 2026 Oct 29;20:1969437. doi: 10.3389/fncel.2026.1969437 (PMC13754709; doi:10.3389/fncel.2026.1969437)
Supplement: Supplementary file 1 [file Table_1.docx]

**Supplementary Table S1. Contextual and indirect experimental evidence relevant to thalamic remodeling after SCI.**

| **Study** | **Model and time point** | **Pain-related phenotype** | **Thalamic target and approach** | **Key thalamic finding** | **Interpretation and evidence boundary** |
| --- | --- | --- | --- | --- | --- |
| **Contextual SCI evidence: general remodeling, at-level phenotypes, or non-thalamic supraspinal endpoints (Category 4)** | | | | | |
| **Mandwie et al. (2022)**  **Category 4** | Female rats; mild T10 contusion; 24 hours. | Pain behavior was not a primary endpoint. | Gross lateral thalamus; GFAP and Iba1 transcript/protein assays. | Thalamic GFAP decreased, whereas Iba1 was not significantly changed. | Acute, cell-marker-specific response without pain phenotyping or nucleus-level resolution. |
| **Gerke et al. (2003)**  **Category 4** | Rats with contusive SCI; 7 days; allodynic and non-allodynic subgroups. | Predominantly at-level flank allodynia-like behavior. | VPL single-unit recording with brush stimulation. | Oscillatory spontaneous firing increased across SCI groups; brush-evoked and afterdischarge responses were greater mainly in the allodynic subgroup. | Separates general SCI dysrhythmia from phenotype-linked evoked responses, but the phenotype was at level and thalamic causality was not tested. |
| **Hains et al. (2006)**  **Category 4** | Male rats; T9 contusion; VPL recording at 28 days. | Behavioral linkage is indirect in the summarized experiment. | VPL single-unit burst analysis; lumbar intrathecal Nav1.3 antisense. | SCI altered burst structure, including fewer spikes and shorter bursts with longer intra- and inter-burst intervals; antisense partly normalized timing. | Shows Nav1.3-related temporal coding changes, but lumbar delivery is not thalamus-specific and concurrent below-level behavior is not the central endpoint. |
| **Brown et al. (2022)**  **Category 4** | Male and female mice; unilateral C5-C6 contusion; 14 and 35 days. | At-level forepaw mechanical and thermal evoked hypersensitivity. | Activity-dependent labeling in thalamus, PAG, and cervical dorsal horn. | Labeled neurons increased in thalamus and decreased in PAG, with increased dorsal-horn projection and excitatory populations. | Maps distributed recruitment after cervical SCI; thalamic nucleus, firing properties, and causal contribution were not resolved. |
| **Hubscher and Johnson (2006)**  **Category 4** | Male rats; severe T8 contusion; approximately 30 days. | At-level somatic and visceral stimulation; new tactile responses mainly accompanied at-level allodynia-like behavior. | Neural response mapping across ventral and posterior thalamic regions. | SCI produced spatially heterogeneous changes and newly emergent thalamic responses to somatic and visceral stimulation. | Demonstrates sensory remapping, but the principal phenotype was at level rather than below-level neuropathic pain. |
| **Castany et al. (2023)**  **Category 4** | Female mice; mild T8-T9 contusion; outcomes through 12 weeks. | Evoked hindpaw hypersensitivity and later affective/nonreflexive pain-related behaviors. | Medial prefrontal and distributed supraspinal glial and CX3CL1/CX3CR1 measures; no thalamic neuronal endpoint. | Chronic affective changes accompanied medial prefrontal glial and chemokine-signaling alterations. | Provides distributed supraspinal inflammatory context rather than evidence of a thalamic mechanism. |
| **Indirect biological and baseline-physiology evidence (Category 5)** | | | | | |
| **de Jong et al. (2005)**  **Category 5** | Cultured cortical neurons; glutamate/endangered-neuron paradigm. | Not applicable. | Cell-biological assays of neuronal CCL21 release and microglial migration. | Released neuronal CCL21 induced microglial migration. | Establishes biological plausibility for neuronal CCL21 signaling; no SCI or thalamic model. |
| **de Jong et al. (2008)**  **Category 5** | In vitro neuronal axonal-sorting and transport model. | Not applicable. | Live-cell imaging of CCL21-containing large dense-core vesicles. | CCL21 was preferentially sorted into vesicles transported toward axon terminals. | Supports remote release capacity but does not demonstrate spinal-injury-to-thalamus transport. |
| **Biber et al. (2011)**  **Category 5** | Mice; L5 spinal nerve transection, a peripheral nerve-injury model. | Tactile allodynia after peripheral nerve injury. | Genetic/pharmacological CCL21 manipulation and spinal microglial P2X4 assessment. | Neuronal CCL21 promoted spinal microglial P2X4 upregulation and contributed to tactile allodynia. | Supports a CCL21-microglia-pain pathway outside the thalamus; it cannot establish an SCI thalamic mechanism. |
| **Park et al. (2017)**  **Category 5** | Normal mice; baseline PO physiology. | Not applicable. | PO slice whole-cell recording and GABAA/GABAB pharmacology. | PO neurons showed synaptic and extrasynaptic GABA_A inhibition, while presynaptic GABA_B receptors regulated inhibitory and excitatory transmitter release. | Provides the physiological baseline used to interpret lesion-induced PO changes; no SCI or pain phenotype. |
| **Indirect evidence from selective spinothalamic-tract lesions (Category 5)** | | | | | |
| **Saghaei et al. (2013)**  **Category 5** | Rats; unilateral electrolytic STT lesion; 21-28 days. | Bilateral mechanical withdrawal hypersensitivity; attenuated by estradiol. | Bilateral VPL Iba1 immunoreactivity and estradiol intervention. | VPL Iba1 immunoreactivity increased with a delayed bilateral pattern; estradiol reduced Iba1 and behavioral changes. | Parallel intervention effects do not establish VPL microglial mediation; the selective lesion is indirect for traumatic SCI. |
| **Naderi et al. (2014)**  **Category 5** | Rats; unilateral electrolytic STT lesion; 14 and 28 days. | Mechanical and thermal withdrawal hypersensitivity; attenuated by estradiol. | Ipsilateral VPL microdialysis and HPLC glutamate measurement. | Extracellular VPL glutamate increased and was reduced by estradiol together with behavioral hypersensitivity. | Glutamate source and causal mediation were not resolved; the selective lesion is indirect for traumatic SCI. |

Note. Category 4 denotes contextual SCI evidence without a BLNP-specific thalamic endpoint; Category 5 denotes indirect evidence from selective tract lesions, peripheral nerve injury, baseline physiology, or in vitro studies. All references are listed in the main manuscript.

Abbreviations: BLNP, below-level neuropathic pain; CCL21, C-C motif chemokine ligand 21; CX3CL1, C-X3-C motif chemokine ligand 1; CX3CR1, C-X3-C motif chemokine receptor 1; GABA, gamma-aminobutyric acid; GABAA/GABAB, GABA type A/type B receptor; GFAP, glial fibrillary acidic protein; HPLC, high-performance liquid chromatography; Iba1, ionized calcium-binding adaptor molecule 1; PAG, periaqueductal gray; PO, posterior thalamic nucleus; SCI, spinal cord injury; STT, spinothalamic tract; VPL, ventral posterolateral nucleus.
