# Supplementary material for: Thalamic remodeling underlying below-level neuropathic pain following spinal cord injury: neuroimmune, neurochemical, and circuit-level mechanisms
Source: Front Cell Neurosci. 2026 Oct 29;20:1969437. doi: 10.3389/fncel.2026.1969437 (PMC13754709; doi:10.3389/fncel.2026.1969437)
Supplement: Supplementary file 2 [file Table_2.docx]

**Supplementary Table S2. Human EEG, cortical-network, and contextual evidence relevant to SCI neuropathic pain.**

| **Study** | **Cohort and pain phenotype** | **Modality and thalamic target** | **Main finding** | **Interpretation and evidence boundary** |
| --- | --- | --- | --- | --- |
| **Human structural and functional-network studies without a thalamus-specific pain endpoint** | | | | |
| **Livny et al. (2024)**  **Category 2** | Chronic SCI with central neuropathic pain n=28 and pain-free SCI n=24; pain level was not specified. | Structural and diffusion MRI; callosal, cingulum, and temporal regions. | The pain group had higher fractional anisotropy in the splenium and right posterior cingulum and greater right temporal gray-matter volume. | Distributed structural association without a thalamus-specific endpoint; increased fractional anisotropy should not be overinterpreted. |
| **Wrigley et al. (2009)**  **Category 1** | Complete thoracic SCI with ongoing BLNP n=10, pain-free SCI n=10, and healthy controls n=21. | Tactile-evoked fMRI of primary somatosensory cortex. | The little-finger representation shifted medially after SCI, and shift magnitude correlated with ongoing pain intensity. | Explicit BLNP phenotype but no thalamus-specific endpoint; cortical reorganization and pain were associated, not causally linked. |
| **Li et al. (2020)**  **Category 1** | Incomplete SCI with BLNP n=10, pain-free SCI n=11, and healthy controls n=25. | Insular gray-matter volume and resting-state functional connectivity. | BLNP was associated with stronger posterior/ventral-anterior insular connectivity with cerebellar, occipitotemporal, visual-association, and hippocampal regions. | Pain-specific cortical-network association, but no thalamus-specific endpoint and a small cross-sectional sample. |
| **Park et al. (2023)**  **Category 2** | Incomplete SCI with mixed at-/below-level neuropathic pain n=41 and healthy controls n=32; pain severity was stratified. | Resting-state fMRI graph metrics and fALFF in frontal and posterior-cingulate regions. | Moderate/severe pain was associated with lower left-middle-frontal degree and fALFF; posterior-cingulate metrics correlated with pain intensity. | Retrospective cortical-network evidence without a pain-free SCI comparator or thalamus-specific endpoint. |
| **Kowalski et al. (2023)**  **Category 2** | Chronic SCI n=37; neuropathic pain n=26, including below-level n=18 and at-level n=8; nociceptive pain could coexist. | Resting-state limbic, striatal, thalamocortical, and thalamolimbic connectivity. | Neuropathic-pain intensity was related mainly to limbic/limbic-striatal connectivity, whereas nociceptive-pain intensity related more to thalamic networks. | Useful pain-type comparison, but it does not provide a BLNP-specific thalamic association. |
| **Supportive EEG and electrophysiological studies without thalamic localization** | | | | |
| **Wydenkeller et al. (2009)**  **Category 1** | SCI with BLNP n=17 and SCI without BLNP n=9; deafferentation was considered analytically. | Contact heat-evoked potentials and resting EEG; thalamocortical rhythm inferred. | After adjustment for deafferentation, the BLNP group had a lower 6-12 Hz resting peak frequency; CHEP abnormalities were common in both SCI groups. | Pain-associated EEG slowing without localization of a thalamic generator; CHEP abnormality was not pain-specific. |
| **Vuckovic et al. (2018)^b^**  **Category 2** | Subacute SCI n=31 and healthy controls n=10; groups included existing pain, future pain, and pain-free outcomes. | Longitudinal resting EEG spectral analysis. | Frequency-band differences preceded central neuropathic-pain onset and supported a candidate classification model. | No thalamic localization and no BLNP-only outcome; candidate risk marker requiring external validation. |
| **Anderson et al. (2021)^b^**  **Categories 1-2** | Chronic dataset: BLNP n=10, pain-free SCI n=10, controls n=10; longitudinal dataset: future pain n=10 and pain-free n=10. | Resting EEG Higuchi fractal dimension. | Nonlinear EEG complexity distinguished chronic pain and showed subacute features associated with subsequent pain status. | No thalamic localization; candidate marker without independent clinical validation in this report. |
| **Kumari et al. (2022)^b^**  **Category 2** | Four groups of n=10: healthy controls, chronic SCI-pain, future pain, and pain-free subacute SCI. | Motor-imagery EEG effective connectivity in premotor, insular, and somatosensory-association regions. | Group differences suggested network features present before later neuropathic-pain development. | Task-dependent cortical evidence; no thalamic localization and no isolated BLNP outcome. |
| **Kumari et al. (2023)^b^**  **Category 2** | Related four-group motor-imagery EEG cohort, total n=41; pain level was not isolated. | Frequency-specific EEG lateralization and connectivity measures. | Frequency-band-specific lateralization metrics were associated with later pain occurrence. | No thalamic localization; independent replication is required. |
| **Hasan et al. (2024)**  **Category 2** | SCI with central neuropathic pain n=10, pain-free SCI n=10, and able-bodied controls n=10. | EEG frequency-band network analysis of frontoparietal and sensorimotor systems. | Theta-band frontoparietal changes were more pain-related, whereas beta/gamma sensorimotor changes appeared more SCI-related. | Small cross-sectional groups and no thalamic localization; pain level was not clearly classified. |
| **Wang et al. (2024)**  **Category 1** | SCI with below-level neuropathic pain n=18 and below-level numbness without neuropathic pain n=18. | Resting EEG power and network connectivity. | The pain group showed lower theta/alpha power, higher beta power, and frequency-specific network alterations. | Explicit below-level comparison but no thalamic localization; substantial sex and injury-characteristic imbalance limits interpretation. |
| **Wang et al. (2025)**  **Category 2** | Human SCI-related neuropathic-pain cohort; pain was not separated as at- versus below-level. | EEG analysis of prefrontal high-gamma oscillations and network features. | Prefrontal high-gamma activity and network characteristics were associated with pain symptoms. | Cortical candidate correlate without thalamic localization; sample-specific performance requires independent validation. |
| **Anderson et al. (2025)^bc^**  **Category 2** | Subacute SCI validation datasets: analyzed dataset A n=20 and dataset B n=32; pain outcome assessed at approximately 6 months. | External-dataset validation of EEG network/complexity classifiers. | Classification accuracy decreased to 66.6% in independent validation. | Demonstrates limited cross-sample generalizability; no thalamic localization and no separately reported BLNP-only validation. |
| **Nawaz et al. (2024)**  **Category 2** | Home-based longitudinal pilot in SCI-related central neuropathic pain, n=10; repeated pre/post-medication recordings. | Resting EEG power and reactivity under eyes-open and eyes-closed conditions. | EEG measures varied with pain intensity within participants. | Exploratory state-monitoring evidence; medication can alter EEG independently of pain, and repeated observations are not independent participants. |
| **Contextual evidence for preserved sensory conduction after clinically complete SCI** | | | | |
| **Awad et al. (2020)**  **Category 4** | Chronic clinically complete SCI n=11; no pain-specific outcome. | Functional cortical responses to tactile or noxious stimulation of insensate below-level regions. | Six of 11 participants showed somatotopically corresponding S1 activity during below-level stimulation. | Supports possible preserved somatosensory conduction, but does not show that retained input causes pain or define a thalamic mechanism. |

Note. These studies support clinical phenotyping, candidate electrophysiological measures, distributed-network remodeling, or preserved sensory conduction but do not provide a thalamus-specific pain endpoint. Evidence categories are defined in the main manuscript. All references are listed in the main manuscript.

^b^ Several reports use related longitudinal EEG cohort configurations or reused datasets. They are retained separately for methodological transparency but should not be interpreted automatically as independent replication.

^c^ For Anderson et al. (2025), the table uses the analyzed dataset-B sample stated in the methods; the published abstract reports a different total.

Abbreviations: BLNP, below-level neuropathic pain; CHEP, contact heat-evoked potential; CNP, central neuropathic pain; EEG, electroencephalography; fALFF, fractional amplitude of low-frequency fluctuations; fMRI, functional magnetic resonance imaging; NP, neuropathic pain; S1, primary somatosensory cortex; SCI, spinal cord injury.
